# Assessing levelized cost of electric vehicle recharging in China

**DOI:** 10.1016/j.isci.2024.110690

**Published:** 2024-08-08

**Authors:** Chon Man Tam, I-Yun Lisa Hsieh, Xin Sun

**Affiliations:** 1 Department of Civil Engineering, National Taiwan University, No. 1, Sec. 4, Roosevelt Road, Taipei 10617, Taiwan; 2 Department of Chemical Engineering, National Taiwan University, No. 1, Sec. 4, Roosevelt Road, Taipei 10617, Taiwan; 3 Integrated Research on Energy, Environment and Society (IREES), Energy and Sustainability Research Institute Groningen (ESRIG), University of Groningen, Groningen, the Netherlands

**Keywords:** Energy policy, Engineering, Energy transportation

## Abstract

Electric vehicle (EV) purchasing decisions are significantly influenced by costs. Focusing on China, this research comprehensively examines the levelized costs of EV recharging (including charging and swapping) at the provincial level considering various factors, including charging locations, time of charging, and power levels. Results indicate that the national average EV charging costs, with and without home chargers, amount to 0.973 RMB/kWh and 1.148 RMB/kWh, respectively. Remarkable variations are observed among provinces, with Xinjiang and Shanghai experiencing the lowest and highest levelized cost of EV charging (LCOC), respectively, with disparities of up to 147.26%, primarily attributed to regional discrepancies in electricity prices and vehicle usage intensity. Despite generous capital subsidies, swapping costs remain considerably higher than charging, ranging from 3.780 RMB/kWh to 4.082 RMB/kWh. Additionally, the sensitivity analysis of major parameters, including infrastructure utilization, suggests that levelized EV recharging costs are already more cost-attractive than the fuel costs of comparable gasoline cars at today’s utilization rates.

## Highlights

• Average EV charging cost with home charging access in China is 0.973 RMB/kWh
• Costs vary greatly by province due to electricity prices and vehicle use intensity
• Assessment framework of levelized cost of battery swapping is proposed
• Swapping is always more expensive than EV charging—regardless of charge behaviors

## Graphical abstract

**Figure:**
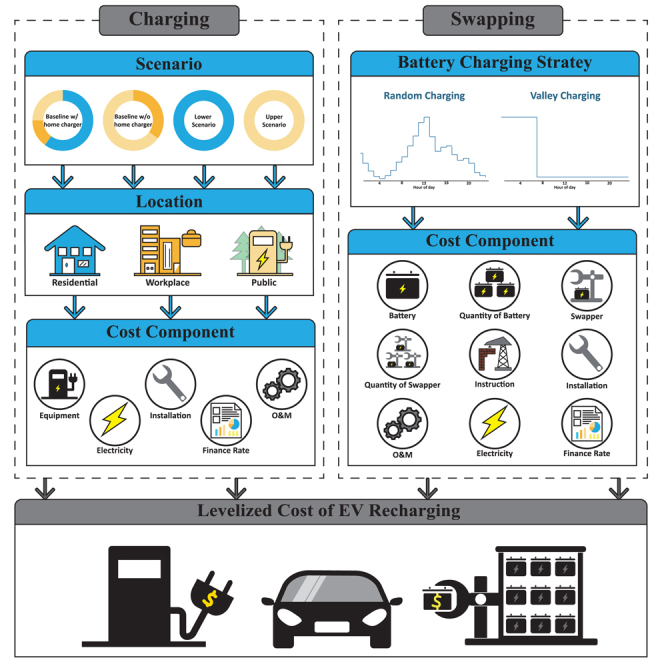

## Introduction

Electric vehicles (EVs) are increasingly recognized as a pivotal development in addressing climate change within the sector of personal transportation. This recognition is underscored by their growing role in reducing dependence on fossil fuels, thereby contributing to a significant increase in global sales since 2020. Worldwide EV sales, which include battery electric vehicles (BEVs) and plug-in hybrid electric vehicles (PHEVs), reached nearly 14 million units in 2023, reflecting an approximate 35% growth compared to the previous year. Notably, China has been a major driver of this growth, fueled by specific government incentives and substantial subsidies, leading to 8.1 million EV sales in 2023—accounting for 60% of the global market.^1^

Consumers are increasingly switching from internal combustion engine vehicles (ICEVs) to EVs, motivated not only by environmental concerns but also by cost considerations.^2^ EVs offer advantages such as lower maintenance costs, higher energy conversion rates, and reduced “fuel” expenses, making them more attractive to consumers.^3^^,^^4^^,^^5^ Researchers have been paying growing attention to the total cost of ownership (TCO) performance of EVs compared to ICEVs, investigating various factors that influence their economic viability.^6^^,^^7^^,^^8^^,^^9^^,^^10^^,^^11^ Previous studies, such as the work by Palmer et al. (2018),^6^ compared the historical TCO of different types of vehicles across multiple regions, highlighting the importance of ownership costs for the adoption of low-emission vehicles. Ouyang et al. (2021)^7^ developed a comprehensive TCO model considering internal and external expenses and projected TCOs for conventional vehicles and EVs in China, indicating the significant impact of incentives on vehicle fleet electrification. Parker et al. (2021)^10^ explored the TCO of EVs and conventional vehicles, taking into account vehicle owners’ usage characteristics and spatial variation, demonstrating that EVs can be more economical than conventional vehicles when considering vehicle miles traveled (VMT). While these studies have provided valuable insights into the total cost of EV ownership, there remains ample scope for further research. Many of these studies have primarily focused on cost comparisons using a single EV charging rate and considered only a limited set of factors that directly or indirectly influence the economics of EV charging.

The cost of charging an EV is influenced by various factors, such as the charging location (residences, workplaces, and public charging), electricity prices at different times (peak and off-peak rates), and the level of electric vehicle supply equipment (EVSE) (e.g., Level 1, Level 2, and DC fast charge).^12^^,^^13^ Previous studies often overlooked the cost variations associated with purchasing and installing EVSE for different charging power levels, potentially leading to biased calculations. Borlaug et al. (2020)^12^ addressed this gap by introducing the concept of the levelized cost of EV charging (LCOC), which considers the variations in EV recharging and usage patterns while distributing all upfront and operating costs over the total energy supplied throughout the EVSE’s lifespan. Their comprehensive study provided a framework for evaluating EV charging costs, focusing on the United States. Although Borlaug et al.'s research significantly advanced our understanding of EV charging economics. While it primarily concentrated on the costs associated with EV charging, it placed less emphasis on other critical factors such as the efficiency of EVSE and transaction costs associated with charging fee payments.

In response, Lanz et al. (2022)^13^ expanded on Borlaug et al.’s model, developing a more comprehensive LCOC approach that incorporated additional factors, including EVSE efficiency and transaction costs. They used this model to assess the LCOC in various European countries, considering diverse equipment options at different charging sites. Lanz et al. also explored the impact of charging facility utilization rates on the LCOC. This advanced model serves as a valuable framework for future research in EV charging economics. Given China’s prominent position as the world’s largest EV market, coupled with the relative scarcity of research on EV charging costs within the country, there is a critical need to investigate the actual charging costs experienced by EV owners across the country. Research focusing on the Chinese market can provide valuable insights into diverse charging scenarios and utilization rates, significantly contributing to understanding the cost-effectiveness and promoting further EV adoption in this key region.

In addition to EV charging infrastructure, battery swapping technology is emerging as an alternative in China. Battery swapping allows EV drivers to quickly replace depleted batteries with fully charged ones, offering a solution closer to the convenience of refueling internal combustion engine vehicles.^14^ This technology boasts several advantages over traditional EV charging, including reduced energy replenishment time, lower upfront purchase costs for EVs, centralized battery management, longer battery lifespan, and the potential to smooth power demand. Currently, battery swapping services are primarily implemented in dense closed taxi ecosystems, where minimizing downtime is crucial for profitability.^15^ Despite the potential benefits of battery swapping, research on its economics is relatively limited compared to EV charging studies.^16^^,^^17^^,^^18^^,^^19^

Hsieh et al.^20^ explored the cost-competitiveness of battery swapping technology compared to EV charging, considering the achievable energy throughput of the fleet network. Their study found that battery swapping, while requiring higher upfront capital costs, can be a cost-effective option on a per-kilometer basis. This study provided valuable insights into the potential economic advantages of battery swapping; however, it did not account for variations in electricity prices. Liang et al.^21^ investigated how different charging strategies and battery swapping station configurations could impact energy use, emissions, and financial feasibility. The study quantified trade-offs between peak load shaving, emission reductions, and profitability, ultimately concluding that battery swapping stations are currently not profitable regardless of the charging strategy. While the focus was primarily on the economic performance of battery swapping, it laid a solid foundation for future research that may compare this method with traditional EV charging. Zhu et al.^22^ compared the economics of energy supply options for heavy electric trucks, including fast charging, supercharging, and battery swapping modes, showing each mode’s system economy under different recharge distances. Although the study was specifically focused on the charging cost competitiveness of heavy-duty trucks, it offered critical findings that can inform broader discussions on energy supply options for various types of EVs.

This study aims to address the limited research on the levelized costs of charging light-duty passenger EVs in China, considering various factors such as charging sites, vehicle use intensity, charging time, power levels, and equipment and installation costs. Building upon the frameworks presented in Borlaug et al.^12^ and Lanz et al. (2022),^13^ this paper adapts the approach to China’s charging context at the provincial level, encompassing an assessment of battery swapping as an alternative recharging option. Given the complexity arising from differences in assumed parameters and data sources across studies, this research meticulously examines the variations in LCOC in China. To gain a comprehensive understanding of the economics surrounding various EV recharging alternatives, this study also includes a comparison of LCOC with the levelized cost of battery swapping (LCOBS). It is essential to note that this study focuses on 30 provinces (Anhui, Beijing, Chongqing, Fujian, Gansu, Guangdong, Guangxi, Guizhou, Hainan, Hebei, Heilongjiang, Henan, Hubei, Hunan, Inner Mongolia, Jiangsu, Jiangxi, Jilin, Liaoning, Ningxia, Qinghai, Shaanxi, Shandong, Shanghai, Shanxi, Sichuan, Tianjin, Xinjiang, Yunnan, and Zhejiang) due to consistent data availability. These provinces represent a significant portion of China’s EV population and economy, offering valuable insights into the overall charging landscape of the country. The exclusion of certain provinces from this study is driven by several regional challenges that could affect the integrity of our analysis. Notably, these provinces feature divergent electricity systems and lack consistent, reliable data on electricity pricing—crucial elements for precise cost analysis. Furthermore, the top-selling vehicle brands and models in these regions differ significantly from those in our study scope, which complicates the uniform application of our analytical framework. These variances impede the uniform application of our analytical framework, risking inaccuracies. By focusing our analysis on the 30 specified provinces, where data standardization and operational conditions are consistent, we guarantee greater reliability and consistency in our results.

## Results

### Variations in levelized cost of EV recharging across provinces in mainland China

The levelized costs of EV charging (LCOC) in mainland China, weighted by the population of provinces (Table S8), are currently 0.973 RMB/kWh and 1.172 RMB/kWh under the baseline scenarios with and without home chargers, respectively. These results are primarily derived from the parameters outlined in Table 1, which provides a detailed account of the assumptions and input values employed in the levelized cost of EV recharging model. In the scenario with home chargers, we assume a charging mix of 60% residential charging (with 80% using Level 2 chargers and 20% using Level 1 chargers^12^), 15% workplace charging, and 25% public charging.^18^ For EV drivers without access to home charging facilities, the charging mix is modeled as 35% workplace and 65% public charging,^18^ reflecting variations in access to personal charging infrastructure among urban and suburban residents. It is worth noting that despite the rapid growth of renewable energy sources in recent years, China still heavily relies on coal power, accounting for approximately 66% of total power generation in 2023.^23^ This continued dependence on coal and regulated power prices contribute to the relatively lower electricity rates and resulting LCOC values for average EV users in China compared to other regions. For EV users with home charging access, the LCOC is 1.046 RMB/kWh (equivalent to 0.161 $/kWh [Careful readers may find differences between the US LCOC numbers presented here and those given in the literature.^12^ These variations are attributed to our recalibration of the results using Equation 1, which ensures a fair and consistent comparison of EV charging costs across regions, considering specific contexts and methodologies used in each study]) in the United States^12^ and ranges from 1.329 to 2.535 RMB/kWh (equivalent to 0.173€–0.330€/kWh) in Europe.^13^

**Table 1:** Governing parameters and the data sources for the calculation of levelized costs of EV recharging in China

Parameter	Baseline case
Charging mix for different sites (Residential (R), Workplace (W), Public (P)) with home charger	60%R, 15%W, 25%P^18^
Charging mix for different sites (Residential (R), Workplace (W), Public (P)) without home charger	0%R, 35%W, 65%P^18^
Discount rate (%)	4.50^24^
Electric vehicle fuel consumption rate (kWh/100 km)	12.84 (see Table S5)
Lifetime (year)	12^25^
Exchange rate for RMB/USD	6.539^26^
Exchange rate for RMB/€	7.683^27^
Residential Charging (R)	
Share of residential AC Level 1 EVSE (L1) and AC Level 2 EVSE (L2)	20% L1, 80% L2^12^
Equipment costs of L2 EVSE (7kW) (RMB)	1,470 (see Figure S2)
Installation cost of L2 EVSE (7kW) (RMB)	750^28^
O&M cost (RMB/yr)	1% of capital costs annually^12^
Annual distance driven (km)	Provincial-level data (see Figure S3)^25^
Electricity price (RMB/kWh)	Provincial-level consumption-weighted average (see Table S4)
EVSE efficiency (%)	L1:100 L2:92^13^^,^^29^
Transaction fees (%)	0^13^
Workplace Charging (W)	
Equipment costs of L2 EVSE (7kW) (RMB)	1,470 (see Figure S2)
Installation cost of L2 EVSE (7kW) (RMB)	750^28^
O&M cost (RMB/yr)	1% of capital costs annually^12^
Daily energy throughput per EVSE (kWh/dy)	32^30^
Electricity price (RMB/kWh)	Provincial-level normal time period commercial electricity price (see Table S6)
EVSE efficiency (%)	92^29^
Transaction fees (%)	0^13^
Public Charging (P)	
Share of different charging rates	1.8% 50 kW, 16.3% 100 kW, 66.6% 150 kW, 15.3% 350kW^30^^,^^31^
Equipment cost (RMB)	62,320 (see Table S7)^32^
Installation cost (RMB)	50% of equipment cost^33^
O&M cost (RMB/yr)	10% of capital cost
Daily energy throughput per EVSE (kWh/dy)	91.5^,^^30^
Electricity price (RMB/kWh)	Provincial-level normal time period commercial electricity price^34^ (see Table S6)
EVSE efficiency (%)	93.1^13^^,^^29^
Transaction fees (%)	0.38^35^
Battery Swapping^21^	
Government capital subsidy rate (%)	30
Annual labor cost (million RMB/yr)	0.8
Lifespan of battery swap stations (year)	10
Annual number of battery pack being swapped in a station (unit)	68,620
Battery pack capacity (kWh)	37.8
Battery price (RMB/kWh)	1551
Random charging strategies	
Number of reversed battery packs	40
Number of chargers	160
Annual electricity cost (million RMB/yr)	2.94
Capital costs (million RMB)	33.74
Annual facility maintenance expenditure (million RMB/yr)	2.11
Valley charging strategies	
Number of reversed battery packs	160
Number of chargers	182
Annual electricity cost (million RMB/yr)	1.90
Capital costs (million RMB)	43.08
Annual facility maintenance expenditure (million RMB/yr)	2.78
Gasoline	
ICEV fuel consumption rate (L/100 km)	8.82^36^
Gasoline price (RMB/L)	5.28–10.24^37^

**Figure 1:**
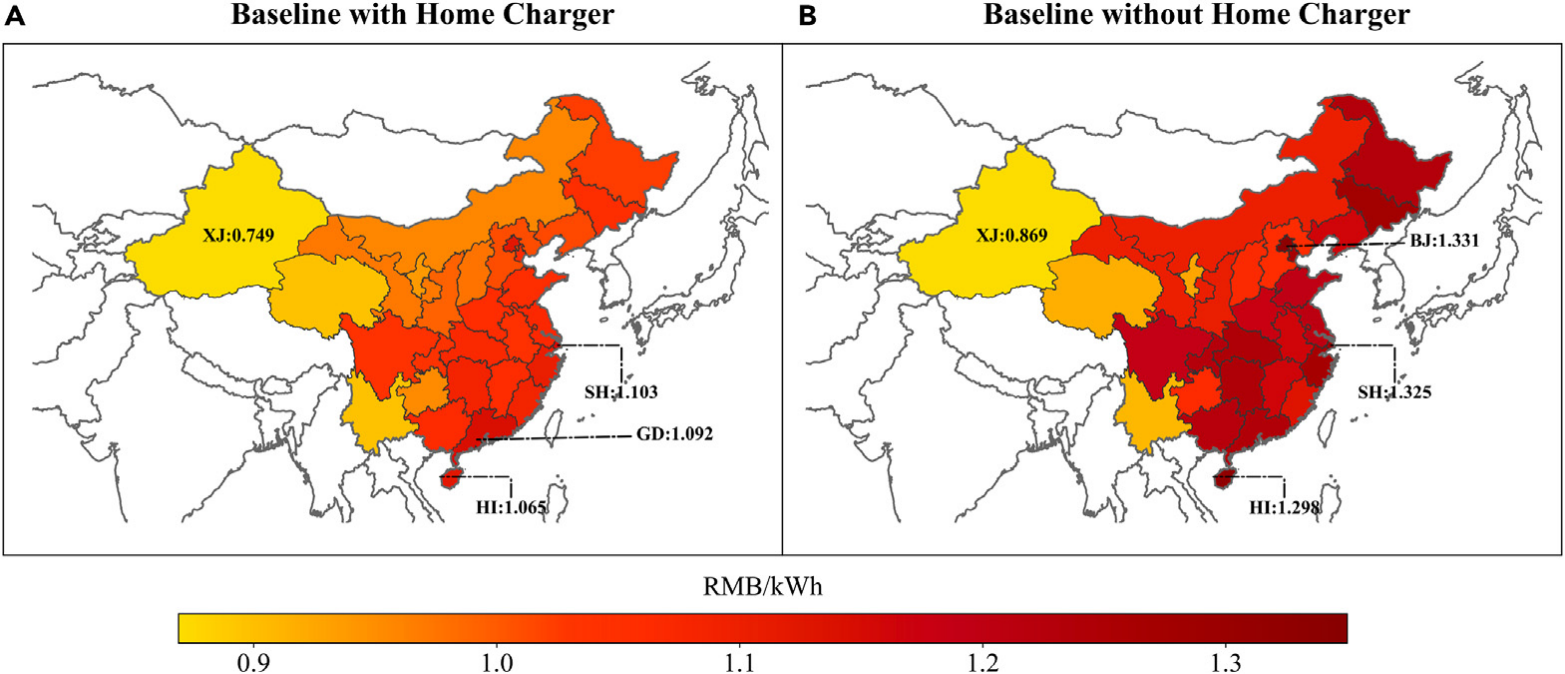
Province-level LCOC variability: (A) with home charger; (B) without home charger Province-level variability in LCOC in the baseline scenario: (A) with home charger; (B) without home charger. Note that only the provinces of China modeled in the present work are shown using colors. Highlights key differences in EV charging costs across provinces, with the highest costs observed in Shanghai, Guangdong, and Hainan when home charging is available, and in Beijing, Shanghai, and Hainan without home charging. Xinjiang consistently has the lowest costs. Note that only the provinces of China modeled in the present work are shown using colors.

Figure 1 illustrates the provincial variations in LCOC for the two baseline scenarios, revealing significant differences in charging costs across China. In the scenario with home chargers, Shanghai, Guangdong, and Hainan are identified as the provinces with the highest EV charging costs. In contrast, in the scenario without home chargers, Beijing, Shanghai, and Hainan incur the highest costs. Notably, Xinjiang consistently presents the lowest charging costs irrespective of the scenario. The discrepancies in LCOC between provinces can be attributed to various factors, including variations in electricity prices influenced by factors such as the costs of power generation (e.g., fuel prices and power plant costs), the state of transmission and distribution infrastructure, local weather conditions, and government regulations. These regional disparities play a crucial role in shaping the overall cost of EV charging for consumers across China.

The disparities between the two baseline scenarios are substantial, particularly for EV users without home chargers In China. On average, when weighted by population, these users face a charging cost of 1.172RMB/kWh. However, if they have the option to charge their eVs at home, this cost can be significantly reduced to 0.973 RMB/kWh—a considerable 16.96% lower. Home chargers provide a significant advantage to EV owners as they eliminate the need to rely on public charging stations and instead allow them to take advantage of low residential electricity prices. Over time, the savings on charging bills would eventually offset the upfront costs of installing Level 2 charging infrastructure at home. The speed at which the cost savings from lower electricity prices can cover the additional capital costs of home chargers is influenced by the vehicle use intensity, as measured by the vehicle kilometer traveled (VKT). VKT is affected by various factors, including the spatial patterns of urban growth, the availability and efficiency of public transport systems, and transport policies. As a result, provincial-level differences in VKT contribute to the wide variations in LCOC across China under the baseline scenario with home charging. In essence, providing home chargers to EV owners can lead to significant cost reductions in charging, making eVs more cost-competitive compared to relying solely on public charging infrastructure.

**Figure 2:**
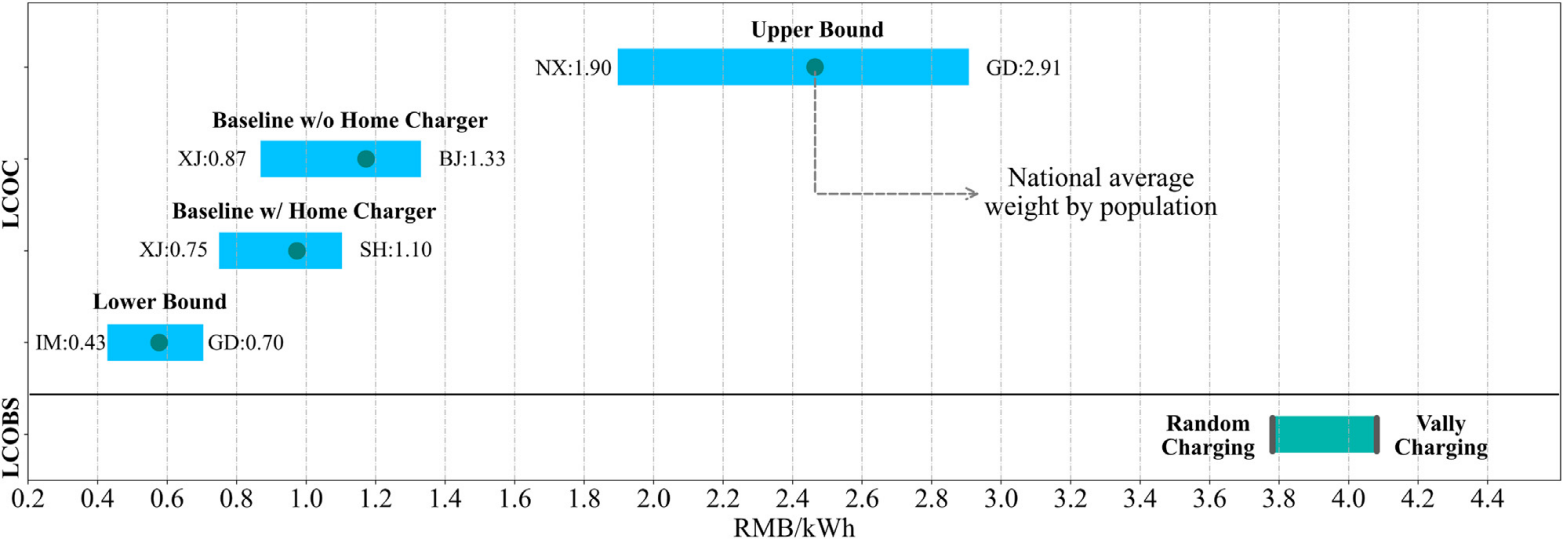
Province-level variability in LCOC for Upper bound, Baseline, and Lower bound scenarios, along with the LCOBS ranges under two different charging strategies (i.e., random charging and valley charging) for swap stations The abbreviations of provinces are provided in Table S6.

Charging behavior, in addition to electricity prices, plays a significant role in influencing the LCOC. Figure 2 provides an overview of the regional variations in LCOC under different charging scenarios, as outlined in the ‘Scenario Designs in EV’ section of the STAR methods and also presented in Table 2. The upper bound scenario exhibits the most extensive variability, with LCOC ranging from 1.896 RMB/kWh in Ningxia (NX) to 2.908 RMB/kWh in Guangdong (GD). On the other hand, the lower bound scenario shows the lowest LCOC variability, ranging from 0.428 RMB/kWh in Inner Mongolia (IM) to 0.704 RMB/kWh in Guangdong. The significant regional variation observed in the upper bound scenario can largely be attributed to the differences in TOU pricing and charging mix across provinces. TOU structures vary significantly among provinces, with commercial peak electricity prices ranging from 0.663 RMB/kWh in Ningxia (NX) to more than double at 1.601 RMB/kWh in Guangdong. Moreover, the charging mix for different charging sites can vary widely among individual EV owners, and this variability has a significant impact on LCOC. Provinces with a higher share of charging performed at public stations would experience amplified differences in charging costs compared to provinces where home charging is more prevalent. As a result, the LCOC analysis tends to favor provinces with lower commercial electricity prices when public charging is the primary charging site.

**Table 2:** Overview of charging scenarios: Baseline, lower bound, and upper bound conditions

Factor	Lower bound	Baseline with home charger	Baseline without home charger	Upper bound
Charging mix	100% residential	60% residential, 15% workplace, 25% public	35% workplace, 65% public	100% public
Residential charging levels	100% L1	20% L1, 80% L2	–	–
EVSE capital costs ($/plug)	$0 (Free)	L2: $1,470 Public: $62,317 (Median)	L2: $1,470 Public: $62,317 (Median)	Public: $125,000 (Upper)
Residential electricity price (RMB/kWh)	Provincial-level consumption-weighted average under stepwise power tariff (SPT) structure (Table S4)	Provincial-level consumption-weighted average under stepwise power tariff (SPT) structure (Table S4)	–	–
Workplace/Public electricity price (RMB/kWh)	–	Provincial-level commercial electricity price during the shoulder time under time-of-use (TOU) rate structure (Table S6)	Provincial-level commercial electricity price during the shoulder time under time-of-use (TOU) rate structure (Table S6)	Provincial-level commercial electricity price during the peak time under time-of-use (TOU) rate structure (Table S6)
Pubic charging power levels	–	1.6 % 50 kW, 10.5% 100 kW, 76.6% 150 kW, 11.3% 350kW	1.6% 50kW, 10.5% 100 kW, 76.6% 150 kW, 11.3% 350kW	100% 350kW

Along with the regional variations in LCOC for EV charging, Figure 2 also presents a comparison of swapping costs under different charging strategies for swap stations in Beijing (BJ). For battery swapping, the potential range of LCOBS varies from 3.780 RMB/kWh for the random charging strategy (i.e., empty batteries charged right after swapping) to 4.082 RMB/kWh for the valley charging strategy (i.e., empty batteries charged only during off-peak hours). When comparing battery swapping with EV charging activities, even with the 30% capital subsidy, battery swapping is currently not cost-competitive. The LCOBS for battery swapping is higher than the LCOC for EV charging by 53.39–554.99% for the random charging strategy and 65.64–607.33% for the valley charging strategy. Although adopting the valley charging strategy allows for peak load shaving and utilizes lower electricity prices during off-peak hours, it requires greater upfront capital costs for additional reserve batteries. As a result, the valley charging strategy still results in higher LCOBS compared to the random charging strategy, as the operating cost savings during off-peak hours cannot fully offset the high upfront battery investments (see Figure S5).

### Sensitivity analysis for the current EV recharging cost

**Figure 3:**
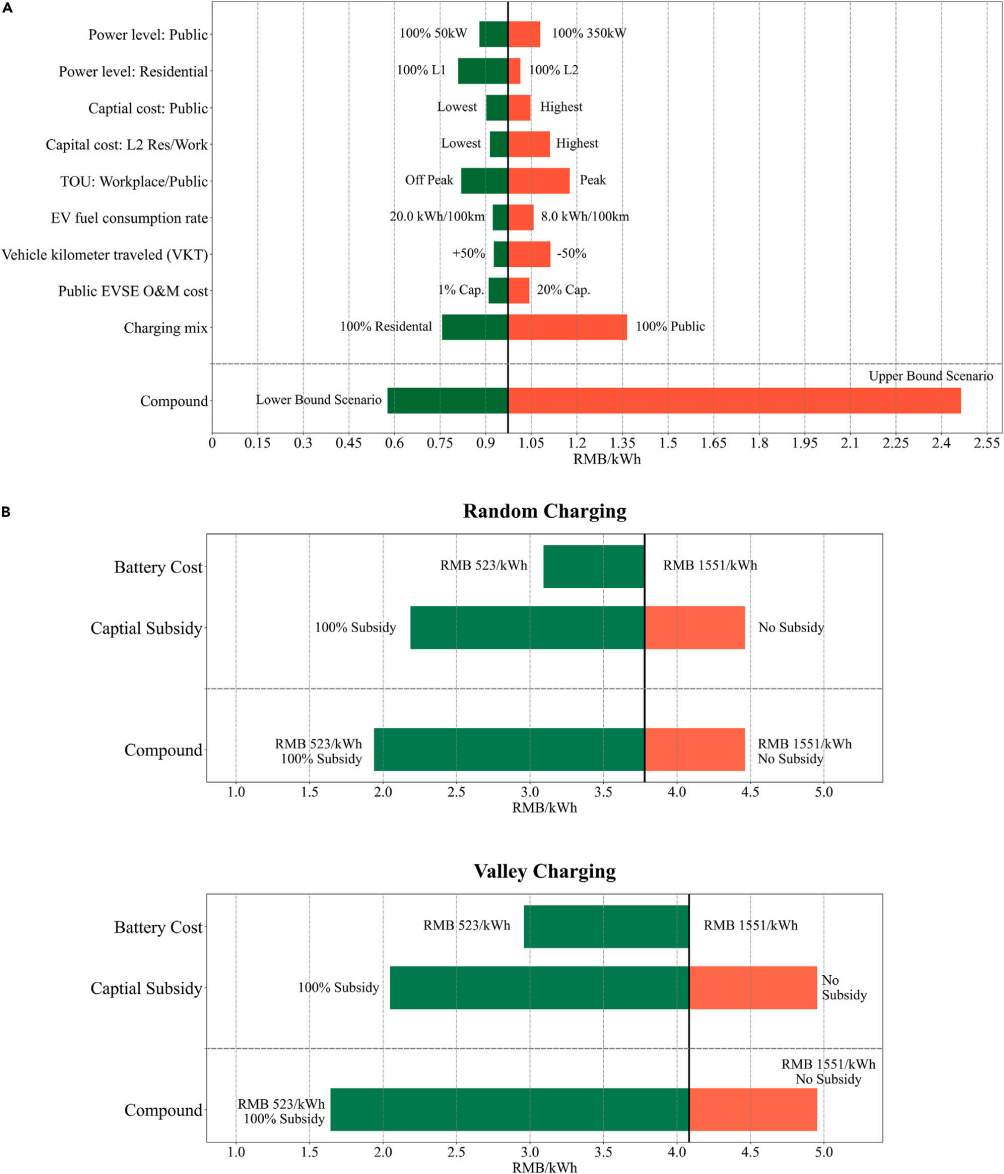
Sensitivity analysis: (A) National LCOC with home chargers; (B) LCOBS for random and valley charging Sensitivity analysis of (A) national LCOC for China’s baseline EVs with home chargers in relation to key factors, including charging time, vehicle use intensity, equipment costs, and charging site mix; (B) LCOBS for random charging and valley charging strategies concerning battery prices and government subsidy. Significant impacts of charging behaviors and site selection on LCOC are highlighted, with the charging mix and vehicle use intensity being the most influential factors. Battery swapping costs are shown to be highly sensitive to battery prices and government subsidies, with valley charging becoming more competitive as battery costs decline.

The levelized costs of EV recharging analyses, as presented in the previous section, are influenced by several factors enumerated in Table 1. To better understand how China’s current national LCOC and LCOBS values are influenced by these factors, we conducted sensitivity analyses, as shown in Figure 3. The parameters considered for the LCOC sensitivity analysis include charging behaviors (e.g., charging site, charging time, and power levels), vehicle use intensity, and upfront equipment costs. For brevity, the sensitivity analysis of LCOC focuses on baseline EV users with home chargers, while the provincial-level sensitivity results are available in Figure S6. On the other hand, the factors examined for the LCOBS analysis are battery pack costs and the government subsidy for high capital investments in battery swap stations. The base values from Table 1 are used to calculate the baseline results (indicated by the black vertical line in Figure 3).

Regarding EV charging costs (Figure 3A), we find that the charging mix, representing the share of charging at different sites, has the most significant impact on LCOC. Charging exclusively at home (i.e., 100% residential charging) reduces the national LCOC by 22.24% (0.216 RMB/kWh), while relying entirely on public charging (i.e., 100% public charging) increases the costs by 40.28% (0.392 RMB/kWh). Vehicle use intensity and charging time are also crucial factors affecting LCOC. Reducing the annual vehicle kilometer traveled (VKT) by 50% to the population-weighted average of 6,013 km leads to a 14.35% (0.140 RMB/kWh) increase in LCOC because the EVSE costs are spread over fewer lifetime kilometers. Conversely, increasing annual VKT by 50% results in a 4.78% (0.047 RMB/kWh) decrease in costs. The time of charging determines the electricity prices (i.e., $C_{\mathrm{el},S}$ in Equation 2) under the TOU pricing structures. If all workplace and public charging occur during off-peak hours, utilizing low TOU rates, the national LCOC would be reduced by 15.84% (0.154 RMB/kWh). Conversely, high peak-hour TOU rates for all non-residential charging would lead to a 20.87% (0.203 RMB/kWh) increase in costs.

The charging levels of EVs, which determine the capital costs of EVSE, are also critical in the LCOC analysis. EVSE with higher power levels typically incurs higher capital costs. Charging exclusively at the highest power level (i.e., 100% 350 kW) at public stations would increase the national LCOC by 10.88% (0.106RMB/kWh), while charging solely at the lowest power level (i.e., 100% 50 kW) would decrease the costs by 9.65% (0.094 RMB/kWh) due to variations in different power-level EVSE capital costs. Charging EVs at home without upgrading to Level 2 EVSE (i.e., 100% Level 1) reduces the LCOC by 16.82% (0.164 RMB/kWh), while paying the additional upgrade costs and relying entirely on Level 2 home charging would only increase the costs by 4.21% (0.041 RMB/kWh).

The capital costs of public DC EVSE have a lesser impact on the national LCOC compared to residential and workplace Level 2 EVSE, with sensitivity ranges of −7.31%–7.60% and −6.08%–14.23%, respectively. This is primarily because the share of total energy replenishment occurring at public charging stations (25%) is relatively lower than that at residential and workplace Level 2 charging combined (63%) in the baseline scenario with home chargers. Additionally, uncertainties in EV fuel consumption rates do not significantly affect the LCOC, ranging from a 5.14% reduction (0.050 RMB/kWh) in the most-efficient EV case to an 8.68% increase (0.084 RMB/kWh) in the worst case. Improving EV fuel consumption rates would reduce the need for charging and result in higher LCOC values because the EVSE capital costs would be spread over fewer lifetime energy supplies. Moreover, the public EVSE operating and maintenance costs also affect the LCOC. Reducing these costs by 90% leads to a decrease in LCOC by 6.507% (0.063 RMB/kWh), whereas an increase in O&M costs by 100% results in an LCOC rise of 7.230% (0.070 RMB/kWh).

At the bottom of Figure 3A, we present the compounded effects of all the major factors on the variations in LCOC. The lower bound represents the most favorable EV charging scenario, which involves the widespread use of Level 1 residential EVSE. On the other hand, the upper bound represents the most unfavorable case, where EVs are charged exclusively at public stations with the most expensive and highest power level EVSE during peak hours. A complete shift from home to public charging amplifies the effects of all the factor interactions, leading to a wide range of LCOC. The best-case scenario reduces the national LCOC by 40.71% (0.396 RMB/kWh), while the worst-case scenario increases the costs by 153.18% (1.491 RMB/kWh).

In terms of battery swapping costs (Figure 3B), we observe that both charging strategies show similar trends: LCOBS decreases as battery costs decline and increases as government subsidies are reduced. Although both strategies are equally sensitive to the capital subsidy, the LCOBS is more responsive to changes in battery costs under the valley charging strategy. As explained in ‘charging strategies for battery swap station’ section of STAR methods, the valley charging strategy requires a higher number of reserve batteries, making its levelized swapping costs improve faster than the random charging strategy when battery costs decrease. The overall results indicate that battery swapping is unlikely to achieve cost parity with EV charging in the foreseeable future, even with declining battery costs and ongoing subsidies. Additionally, we conduct a breakeven analysis to determine the battery cost at which the valley charging strategy would become cost-competitive with the random charging strategy (Figure S7). It is found that when battery costs decline to 504RMB/kWh (a 67.5% reduction), the LCOBS under the valley charging strategy decreases to 3.184 RMB/kWh (a 26.42% decrease), making it more economically favorable compared to random charging.

The sensitivity analysis reveals several key findings. Firstly, home charging remains the most affordable option for recharging EVs due to relatively low residential electricity prices and the capital costs of home L1/L2 EVSE. Secondly, upgrading from L1 to L2 EVSE at home only adds 0.205 RMB/kWh to the charging costs when levelized over 12,025 km annual VKT, offering the benefits of faster charging speed and convenience. Thirdly, EV users without home chargers can minimize costs by taking advantage of off-peak TOU pricing and avoiding charging their EVs during peak hours at workplaces and public charging sites. Under the current TOU rate structures, the average commercial electricity prices during peak hours are 58.40% higher than the shoulder period and even 193.41% higher than off-peak hours. Charging during off-peak hours could result in significant cost savings of up to 30.35% compared to peak-hour charging.

### EV recharging cost as a function of infrastructure utilization

**Figure 4:**
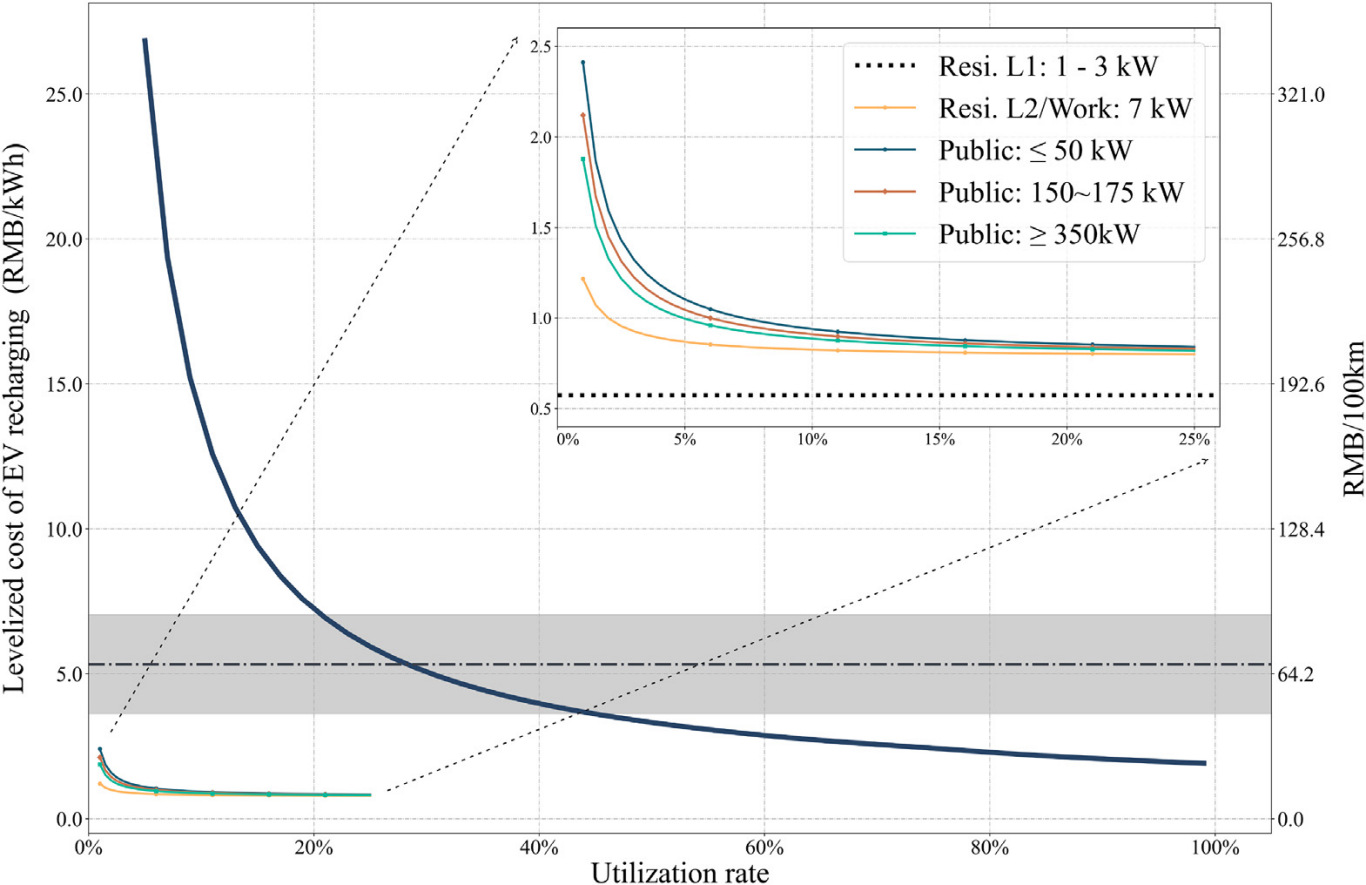
National LCOC with different power levels and LCOBS as a function of infrastructure utilization rate The grey-shaded region indicates the fuel cost ranges of gasoline cars between 2014 and 2022.

In this section, we explore the relationship between EV recharging costs and infrastructure utilization rates. Currently, the charging infrastructure in China is relatively underutilized, with low utilization rates (LCOC baseline: 7.63% for 50 kW, 3.47% for 100–120 kW, 2.34% for 150–175 kW, 1.09% for 350 kW). As the number of EVs on the road is expected to increase in the coming decades, the utilization rates of the charging infrastructure will also grow. It is essential to understand how changing utilization rates impact the national LCOC and LCOBS and how they compare to the fuel costs of conventional ICEVs. We conduct an additional sensitivity analysis to examine the effects of varying utilization rates on the levelized costs of EV recharging, and the results are shown in Figure 4. The utilization rate on the x axis represents the ratio of yearly energy throughput to the maximum achievable energy throughput under the given capacity. Several key observations can be highlighted from the results:

First, Level 1 residential charging, which involves no capital costs, exhibits a levelized cost equal to the residential electricity prices, independent of utilization rates (represented by a horizontal line). Second, for non-residential recharging options, the levelized costs decrease exponentially as utilization rates increase. Higher utilization leads to more efficient use of the fixed capital costs, resulting in lower levelized costs. Third, among different public EV charging rates, DC charging with higher power levels has lower LCOC values at given utilization rates due to its ability to accommodate more energy throughput. Fourth, comparing LCOC to LCOBS, significantly higher utilization rates are required for battery swap stations to approach their minimum possible levelized costs. Swap stations involve high upfront investments, and breakeven points can only be achieved with high utilization rates. Finally, we compare the EV recharging costs to the fuel costs of conventional gasoline cars in China by converting both LCOC/LCOBS (RMB/kWh) and gasoline prices (RMB/L) to a common unit of measurement—RMB per 100 km, considering the fuel consumption rates of EVs and ICEVs as 12.84 kWh/100 km and 8.82 L/100 km, respectively. Our analysis reveals that as long as utilization rates exceed 21–43%, depending on the charging strategies for swap stations, levelized swapping costs will be lower than fuel costs.

## Discussion

This study delivers an extensive analysis of the levelized costs of recharging for EVs in China, showcasing the newly developed LCOBS model. This innovative model analyzes various swapping cost factors and represents a substantial advancement in the study of EV recharging economics. Our evaluation encompasses diverse considerations including the choice between charging or swapping, the timing of recharging (peak vs. off-peak hours), and the location of recharging sites (home, workplace, or public). By investigating the average levelized costs across different user preferences and locations within China, we conduct an in-depth sensitivity analysis to determine the impact of varying charging behaviors on overall costs. Moreover, we explore the relationship between EV recharging costs and infrastructure utilization rates, comparing these costs to those of conventional gasoline vehicles. This comprehensive approach not only illuminates the economic viability of various EV recharging options but also provides valuable insights into the broader implications for the transportation sector and a thorough evaluation of the total cost of EV ownership.

The analysis of levelized costs of EV recharging reveals several prominent patterns. First, the national average LCOC for an average EV user with home chargers in China is 0.973 RMB/kWh, which is significantly lower compared to the two largest EV markets—the United States and Europe, being 6.98% and 26.79–61.62% lower, respectively. This difference can be attributed to China’s relatively lower electricity prices. Second, EV users with home charging access enjoy lower LCOC due to their ability to utilize more affordable household electricity. Conversely, those without home chargers face an average increase of 20.45% in non-residential charging costs, primarily due to higher EVSE capital costs and more expensive commercial electricity. Third, the high capital investments required for swap stations lead to LCOBS values consistently higher than LCOC, irrespective of charging behaviors. For instance, in Beijing, LCOBS is 65.67–607.45% higher than LCOC under different charging scenarios with the random charging strategy.

In addressing regional variations in electricity prices and vehicle use intensity, our analysis identifies significant disparities in the LCOC across provinces. For instance, disparities as high as 148.42 and 157.68% have been observed between baseline EV users with and without home chargers, respectively. These variances largely stem from regional differences in electricity tariffs and vehicle usage patterns, which in turn influence the economic viability of EV adoption in different areas. Regions such as Guangdong, Shanghai, and Beijing, where higher charging prices prevail, may require targeted governmental interventions to lower barriers to EV adoption. These could include subsidies for EV purchases, reduced tariffs for electricity used in EV charging, and financial support for the expansion of charging infrastructure. Such measures would not only make EVs more affordable but also improve the overall service quality of charging facilities. Conversely, provinces like Xinjiang, Qinghai, and Yunnan that benefit from lower LCOC offer a different set of opportunities and challenges. Here, the focus might be on maximizing the economic advantages to push for faster market penetration and broader adoption of EVs. Lower operational costs in these regions could be leveraged to attract investments in charging infrastructure, enhancing the accessibility and convenience for EV users. Understanding these regional dynamics is crucial for designing effective policies that support the equitable and sustainable growth of the EV market.

A key finding from the sensitivity analysis is the significant impact of the charging mix on LCOC, as it influences all the costs associated with the primary charging site. For average EV users with home chargers (i.e., 60% residential with 80% being L2 charging), reducing the costs of residential L2 charging would yield the most effective improvement in the current LCOC. While L1 home chargers are usually provided at no additional cost with new EV purchases, opting for L2 chargers could offer faster and more convenient charging options, albeit with a 21.66% increase in upgrade costs. Notably, the growing trend of sharing private charge posts in the Chinese sharing economy has demonstrated substantial potential for cost savings (20%–50%) for charger owners due to increased utilization,^38^ consistent with the findings of this study that higher utilization rates lead to lower levelized charging costs. For average EV users without home chargers (i.e., 35% workplace and 65% public charging), the flexibility in scheduling non-residential charging significantly impacts the LCOC. Taking advantage of TOU tariffs and charging during off-peak hours can amplify cost savings; the difference in LCOC between 100% off-peak and peak charging is approximately 30.37%.

The analysis of battery swapping reveals that the adoption of the valley charging strategy, while beneficial in reducing peak demand, results in 27.67% higher capital costs due to the need for additional reserve batteries. Despite currently being more expensive, LCOBS with the valley charging strategy is projected to improve at a faster rate as battery costs decline. Once battery costs reach 838 RMB/kWh (46% reduction), swapping with valley charging will achieve cost parity with random charging. However, the sensitivity analysis suggests that LCOBS is not likely to achieve parity with LCOC in the foreseeable future, even with reductions in battery costs to $80/kWh (the target set by the United States Department of Energy^39^). Under the existing subsidy schemes, LCOBS remains 39.16–42.49% more expensive than the upper bound scenario of EV charging. It is essential to note that the study does not consider opportunity costs associated with recharging times. While EV charging can take at least 30 min (up to more than 10 h) for 80% energy replenishment, battery swapping requires only a few minutes to replace depleted batteries with fully charged ones. The cost competitiveness among different EV recharging options could change significantly when considering the opportunity costs related to long charging times.

This study provides actionable insights into the levelized costs of EV recharging and battery swapping, offering a practical guide for various stakeholders. To reduce the LCOC, policymakers should focus on enhancing EVSE infrastructure. Recommended strategies include subsidizing residential Level 2 chargers, promoting the sharing of private charge posts to boost utilization, and implementing TOU tariffs to encourage off-peak charging. These initiatives are projected to significantly reduce costs. Additionally, to counter the higher initial costs of battery swapping, we advocate for advances in battery technology and financial incentives for valley charging techniques. It is crucial for operators to strategically position charging and swapping facilities based on regional demand to optimize efficiency and effectiveness. Implementing these measures will not only lower EV recharging costs but also support the growth of the EV sector and facilitate the development of vital charging infrastructure.

In the next decade, the widespread adoption of EVs will play a crucial role in decarbonizing China’s road transport sector. However, this transition is contingent on the development of a robust recharging infrastructure, which, in turn, depends on the number of EVs on the roads—a classic chicken-and-egg dilemma. Uncertainties regarding future demand challenge long-term investments in recharging networks for stakeholders like automakers and the energy industry. Effective coordination between governments and various EV ecosystem players is essential to overcome these deployment challenges. This study’s findings and suggestions enable informed decisions on EV adoption and infrastructure development. By understanding these costs, stakeholders can collaboratively drive EV growth and optimize recharging infrastructure, contributing to the successful and sustainable integration of EVs into China’s transportation ecosystem.

### Limitations of the study

First, our model does not include land costs. While we assume that residential and workplace EVSE installations occur on private premises without incurring land rental costs, the scenario for public EVSE operators can be quite different. They may engage in various business strategies that involve rental locations, which could significantly impact the overall cost structure. Therefore, incorporating land costs is essential for a comprehensive evaluation of public EVSE economics. Additionally, the assumption that one residential EVSE charges only a single EV throughout its lifespan may not hold as households increasingly adopt multiple EVs. This could lead to higher utilization of the installed residential EVSE, altering the dynamics of capital cost amortization and potentially leading to an overestimation of levelized EV recharging costs for such households. Moreover, the study captures the current levelized costs of charging and battery swapping based on present-day technologies for batteries, EVSE, and battery swap stations. Although sensitivity analyses are conducted to consider technological advancements, the evolving nature of these technologies and their impact on costs might not be fully represented in our current model. As such, it is crucial that future studies update cost models to reflect technological progress for more accurate forecasting. Furthermore, this study relies on multiple assumptions to model charging behaviors and swapping demand patterns, which may not fully capture the actual scenarios across various provinces. Specifically, our upper and lower-bound scenarios, while useful for representing extreme cases and analyzing swap station utilization, may not adequately reflect the diverse user behaviors and infrastructure realities. To address this, future research should focus on gathering and analyzing detailed regional data. This will allow for more accurate and location-specific insights, ultimately enhancing the applicability and relevance of the findings to real-world conditions. The reliance on data from a single source for calculating LCOBS also poses a limitation. This source, while providing detailed cost breakdowns, does not reflect the competitive market dynamics or the diversity of operational strategies among battery swap station operators. To overcome this, acquiring a more varied dataset that includes different types of battery swap stations will be critical for understanding the broader cost implications. Lastly, this study focuses on the minimum price under the assumption of operator breakeven, which may not align with the actual consumer prices at public charging and swap stations. These prices could vary based on the specific business models and pricing strategies employed by operators. Investigating how these business models influence recharging costs will yield deeper insights into the pricing strategies’ impact on the overall cost of recharging services.

## Resource availability

### Lead contact

Further information and requests for resources should be directed to and will be fulfilled by the Lead Contact, I-Yun Lisa Hsieh (iyhsieh@ntu.edu.tw).

### Materials availability

The availability of data and code generated through this analysis are outlined in the data and code availability statement.

### Data and code availability

• The data used in this paper have been deposited at https://github.com/NTU-E3group/LCOC-and-LCOBS-model and is publicly available as of the date of publication. DOIs are listed in the key resources table.
• All original code has been deposited at https://github.com/NTU-E3group/LCOC-and-LCOBS-model and is publicly available as of the date of publication. The DOI is listed in the key resources table.
• Any additional information required to reanalyze the data reported in this paper is available from the lead contact upon request.

## Acknowledgments

This work was supported by the 10.13039/100020595National Science and Technology Council, Taiwan (NSTC 113-2621-M-002-006).

## Author contributions

Conceptualization, C.M.T. and I.-Y.L.H.; Methodology, C.M.T. and I.-Y.L.H.; Formal Analysis, C.M.T.; Investigation, C.M.T. and X.S.; Writing – Original Draft, C.M.T.; Writing – Review and Editing, I.-Y.L.H. and X.S.; Funding Acquisition, I.-Y.L.H.; Resources, I.-Y.L.H. and X.S.; Supervision, I.-Y.L.H. and X.S.

## Declaration of interests

The authors declare no competing interests.

## STAR★Methods

### Key resources table

**Table:** 

REAGENT or RESOURCE	SOURCE	IDENTIFIER
**Deposited data**		

EVSE’s cost parameters, and energy efficiency	This paper	N/A
China’s provincial-level electricity price	National Bureau of Statistic.	http://www.stats.gov.cn/tjsj/
Swap station cost parameters	Liang et al.^21^. Configuration and system operation for battery swapping stations in Beijing. Energy 214, 118883.	https://doi.org/10.1016/j.energy.2020.118883.

**Software and algorithms**		

Python	Python Software Foundation	https://www.python.org/
Code for model building and evaluation	GitHub repository	https://github.com/NTU-E3group/LCOC-and-LCOBS-model

### Method detail

#### Levelized cost overview of electric vehicle recharging

EVs offer greater flexibility for recharging compared to internal combustion engine vehicles (ICEVs), with multiple charging options available at diverse locations. This study analyzes the charging costs of EVs at three major sites: home, workplace, and public charging stations. The assessment considers various factors, including equipment and installation costs, operating and maintenance costs, and electricity prices, to understand the variations in EV charging costs at different locations. Additionally, the study explores battery swapping, an emerging alternative to EV charging gaining popularity in China. Battery swapping allows drivers to replace depleted batteries with fully charged ones quickly, resembling the refueling process for ICEVs. To provide a comprehensive comparison, the levelized cost of EV recharging is thoroughly analyzed among different charging options.

Figure S1 provides an overview of the EV recharging cost assessment approach utilized in this study. Firstly, we analyze the LCOC for each EV charging site using Equation 1. Subsequently, the individual site costs are combined, taking into account the charging mix (${CM}_{s}$) as weighting factors, to calculate a combined levelized cost of charging ($LCOC_{comb})$ as represented by Equation 2. Table 1 presents a detailed list of parameters employed in calculating the levelized cost for EV recharging in China.

(Equation 1) $$LCOC_{s}=\left(\frac{C_{capital,s}+\sum_{i=1}^{N}\frac{C_{O\&M,s,i}}{{\left(1+dr\right)}^{i}}}{\sum_{i=1}^{N}\frac{E_{i,s}}{{\left(1+dr\right)}^{i}}}+\frac{C_{el,s}}{\eta }\right)\times \left(1+{TF}_{s}\right)$$

(Equation 2) $$LCOC_{comb}=\sum_{s}{CM}_{s}\times LCOC_{s}$$

In these equations, ${CM}_{s}$ represents the charging mix, which is the fraction of total energy replenishment occurring at charging site s. $LCOC_{s}$ is the levelized cost of charging at charging site s, which depends on various factors:

$C_{capital,s}$: Initial capital costs of the EVSE at site s.

$C_{O\&M,s,i}$: Recurring operating and maintenance costs of the EVSE at site s in year i.

$C_{el,s\;}$: The electricity prices charged by electric power companies at charging site s.

$E_{i,s\;}$: The energy throughput during the EVSE lifespan at charging site s in year i.

N: The EVSE lifespan. Due to the lack of specific data, we assume N to be equivalent to the average vehicle lifespan in China, which is 12 years.^25^

dr: The discount rate. For cost calculations over the EVSE lifespan, we assume a discount rate of 4.5%. This rate is in line with the current Chinese central bank’s interest rate for long-term loans (i.e., more than 5 years).^24^

η: The EVSE charging efficiency.

S: Charging sites where EVs are recharged, which include residential, workplace, and public charging. These will be elaborated upon in further detail later in this section.

${TF}_{s\;}$: Transaction fees at charging site s, which represent the percentage of the payment amount levied by the financial company for the operator upon the completion of the electric vehicle charging process and the initiation of the transaction by credit card or other means.

#### LCOC: Residential charging

Residential charging is the most popular and cost-effective method for EV owners, as it allows convenient overnight charging at lower residential electricity prices. The LCOC for residential charging is calculated using Equation 2, with the yearly energy throughput estimated from Equation 3.

(Equation 3) $$E_{i,res}=VKT\times FCR\times CM_{res}$$

where VKT is the annual vehicle kilometers traveled (see Figure S3)^25^; FCR is the EV fuel consumption rate derived from the best-selling EV models in China in 2023 (see Table S5); $CM_{res}$ is the fraction of EV charging that occurs at home, which on average is 60% in China.^18^

For residential charging, Level 1 (1–3 kW) chargers are typically provided for free to new EV owners in China. However, many EV owners prefer faster charging speeds and opt to upgrade their home charging facilities to Level 2 chargers, incurring additional costs. $C_{capital,res}$ is the upfront capital cost for a Level 2 (7 kW) household EVSE, including equipment and installation costs. Equipment cost data is collected from multiple dominant online commerce platforms^40^^,^^41^ (see Figure S2). Based on real-world Level 2 charger transaction data (6,248 cases), equipment costs vary significantly, ranging from the cheapest at 600 RMB to the most expensive at 4,965 RMB, with a median equipment cost of 1,470 RMB (used as the baseline case). Installation costs are assumed to be 750 RMB.^28^ Annual operating and maintenance costs are assumed to be 1% of the initial capital.^12^ The EVSE charging efficiency (η) is assumed to be 100% for Level 1 charging^13^ and 92% for Level 2 charging.^29^ Furthermore, as residential charging is considered a private site, no financial transaction fees are incurred. The residential electricity prices are derived from the stepwise power tariff mechanisms and the monthly or annual electricity consumption at the provincial level.^42^ Consumption-weighted averages of residential marginal electricity prices are detailed in Table S4.

#### LCOC: Workplace charging

As the adoption of EVs increases, private businesses and other organizations are installing Level 2 EV chargers, mostly at 7 kW in China, for their employees. The workplace LCOC is calculated using Equation 2, with the yearly energy throughput ($E_{i,wor}$) estimated using Equation 4:

(Equation 4) $$E_{i,wor}=E_{daily}\times D$$

where the daily energy throughput ($E_{daily}$) is assumed to be 32 kWh supplied by workplace EVSEs, operating for 4.5 h per day.^30^ The charging day (D) represents the total number of weekdays in a year, which is 261. The assumed EVSE lifespan (N) is 12 years. The capital costs at workplaces ($C_{capital,wor}$) are the same as those for Level 2 EVSEs in households (see Figure S2). The EVSE efficiency (η) for Level 2 charging is assumed to be 92%.^29^ Since workplace charging is considered private, the analysis does not account for any financial transaction fees. Under time-of-use (TOU) rates, the price of commercial electricity varies depending on the time of day when it is consumed. As the working hours are mostly from 9:00 to 18:00, we adopt the rates during the shoulder periods as the electricity price of workplace charging ($C_{el,wor}$).

#### LCOC: Public DC charging

While home charging is the preferred method for EV owners, public charging infrastructure is essential for residents without access to home chargers. In this study, we focus on DC charging at a rate of greater than 50 kW, which is the most commonly charged power level at public charging stations in China.^30^^,^^31^ We consider typical DC charging rates of 50 kW, 100 kW, 150 kW, and 350 kW and calculate the corresponding LCOC using Equation 2. To determine the charging mix weighted average LCOC for public DC charging, we use Equation 5:

(Equation 5) $$LCOC_{public}=\sum_{P}{PCM}_{p}\times LCOC_{public,p}$$

where ${PCM}_{p}$ is the public charging mix—the fraction of DC charging at different power rates p.^12^ The capital costs of public DC EVSEs ($C_{capital,public}$) include equipment and installation costs, which vary by power levels. Generally, higher power chargers are more expensive (see Table S7); the median values range from 22,690 RMB/plug at 50 kW to 107,123 RMB/plug at 350 kW. The yearly energy throughput ($E_{i,pub}$) for public charing is estimated using Equation 4, where the daily energy throughput ($E_{daily}$) is assumed to be 91.5 kWh,^30^ and the charging day (D) is 365.

To account for the operational complexities at public charging stations, including the management, maintenance, and labor fees, we conservatively assume 10% of upfront capital costs as O&M costs. Given the scarcity of detailed data on these costs, this study employs sensitivity analysis to explore the potential variability and its implications for the LCOC. This approach not only aligns with industry practices but also highlights areas where further research could enhance understanding and precision in future analyses. In the case of DC fast charging, the analysis considers the efficiency of both the transformer and the charger. The transformer is assumed to have a 98% efficiency,^13^ while the DC EVSE is assumed to have a 95% efficiency,^29^ resulting in an overall efficiency (η) of 93.1% (i.e., 98% × 95%). Additionally, a surcharge of 0.38% on the total LCOC is assumed to incorporate the transaction fees (TF) associated with financial transactions.

The electricity prices for public DC charging vary depending on factors such as charging time (under TOU rates), station size, and capacity. In the baseline scenario, we assume EV charging occurs during shoulder hours, and we explore the impacts of TOU pricing (i.e., high prices in peak times and low prices in off-peak times) on LCOC using sensitivity and scenario analyses. Land costs are not considered in the study due to the variation in land prices across China and unknown public EV charging business models. Additionally, returns on investments in public charging infrastructures are assumed to be zero, meaning that operators would charge customers at their breakeven prices.

#### Levelized cost of battery swapping (LCOBS): Public battery swapping

In contrast to the relatively long charging times associated with Level 2 chargers, battery swapping technology offers a promising solution to address the charging conundrum by significantly shortening the charging process from hours to minutes. This advantage becomes particularly valuable in high-density urban areas like China. As the Chinese government started providing subsidies to battery-swap business models, battery swapping technology has gained traction and is rapidly expanding in the country. Notably, Beijing has emerged as a major hub for battery swapping, boasting the highest concentration of swap stations in China and providing extensive swapping services to city fleets.^31^ To assess the economics of public battery swapping, this study utilizes operation data from the swapping system in Beijing.^21^ The levelized cost of battery swapping (LCOBS) for public battery swapping is calculated using Equation 6, with the yearly energy throughput estimated from Equation 7:

(Equation 6) $$LCOBS=\frac{C_{capital,BS}+\sum_{i=1}^{N_{BS}}\frac{C_{O\&M,BS,i}}{{\left(1+dr\right)}^{i}}}{\sum_{i=1}^{N_{BS}}\frac{E_{i,BS}}{{\left(1+dr\right)}^{i}}}$$

(Equation 7) $$E_{i,BS}=n_{sb}\times {Cap}_{sb}$$

where $C_{capital,BS}$ represents the subsidized capital costs of a battery swapping station, which takes into account the government capital subsidy deducted from the total costs of constructing the station, acquiring swappable batteries, and installing the charging facility; $C_{O\&M,BS,i}$ includes the annual operating and maintenance costs, covering regular maintenance expenses, labor costs, and annual electricity costs for charging the swappable batteries; dr is the discount rate; $N_{BS}$ is the lifespan of a battery swap station; $n_{sb}$ is the annual number of batteries being swapped in a station; ${Cap}_{sb}$ is the capacity of a swappable battery. Based on Liang et al. (2021),^21^ a battery swap station typically comprises 40 swappable battery packs, each with a capacity of 37.8 kWh. The initial capital investment required for such a station amounts to approximately 44 million RMB, with the government providing a 30% capital subsidy.^21^ On average, each swap station provides 194 swaps per day, and the electricity costs are determined by the hourly variations in swapping demand (see Figure S4) under the TOU rate structure.^21^ Similar to the case of public DC charging, this study does not take into account land costs and returns on investments when calculating the LCOBS.

#### Scenario designs in EV charging

Scenario designs in EV charging are essential to capture the diverse charging behaviors and assess the average costs of EV charging in China. In this study, we develop two baseline scenarios to analyze the average costs of EV charging in China. The first baseline scenario, referred to as the “Baseline with Home Charger,” assumes a charging mix of 60% residential, 15% workplace, and 25% public charging. Within the residential charging mix, it is assumed that 80% of the charging relies on Level 2 chargers.^18^ This scenario reflects the typical charging distribution when EV owners have access to home chargers. Considering that a significant number of EV drivers in China do not have access to residential parking and home charging facilities,^18^^,^^43^ we design a second baseline scenario, termed the “Baseline without Home Charger.” In this scenario, we assume that 35% of EV charging occurs at workplace sites, while 65% takes place at public charging stations. Home charging is not considered in this scenario, reflecting the situation where EV owners primarily rely on workplace and public charging options.

To capture the diversity of charging behaviors and their corresponding costs, we create two additional scenarios. The “Lower Bound Scenario” represents the charging condition with the lowest possible charging costs, assuming that 100% of residential charging takes place using Level 1 chargers. L1 chargers have lower charging speeds and are generally less expensive, making this scenario the most cost-effective charging option. On the other end of the spectrum, the “Upper Bound Scenario” represents the condition with the highest possible charging costs. In this scenario, we assume that all EV charging occurs exclusively at the most expensive option, which is 100% at 350-kW public DC charging stations. Table 2 presents a comprehensive overview of the different scenario designs, outlining the charging mixes and key cost factors considered in China.

#### Charging strategies for battery swap station

Under the TOU tariff structures, the cost of recharging swappable EV batteries can vary significantly based on the time of charging. To explore the impact of different charging strategies, we evaluate two approaches for recharging empty batteries to meet the hourly swapping demand (see Figure S4), as proposed by Liang et al.^21^ The first strategy is random charging, where batteries are charged immediately after being swapped—which is the most common approach used in existing swap stations. The second strategy is valley charging, which involves charging all the swapped-out batteries during off-peak hours. The valley charging strategy offers the advantage of peak load shaving, resulting in potential cost savings. However, it requires higher capital investments compared to random charging. Specifically, the valley charging strategy necessitates an additional 300% reserve batteries (160 versus 40 battery packs) and 13.75% more chargers (182 versus 160 chargers)^21^ in a swap station to accommodate charging during limited off-peak hours when depleted batteries can be recharged.
