# Supplementary material for: Assessing levelized cost of electric vehicle recharging in China
Source: iScience. 2024 Aug 8;27(9):110690. doi: 10.1016/j.isci.2024.110690 (PMC11406093; doi:10.1016/j.isci.2024.110690)
Supplement: Document S1. Figures S1–S7 and Tables S1–S8 [file mmc1.pdf]

iScience, Volume *27*

Supplemental information

Assessing levelized cost of electric vehicle recharging in China

Chon Man Tam, I-Yun Lisa Hsieh, and Xin Sun

Supplemental information

S1. Overview of the combined levelized cost of charging ($LCOC_{comb}$) assessment approach in China.

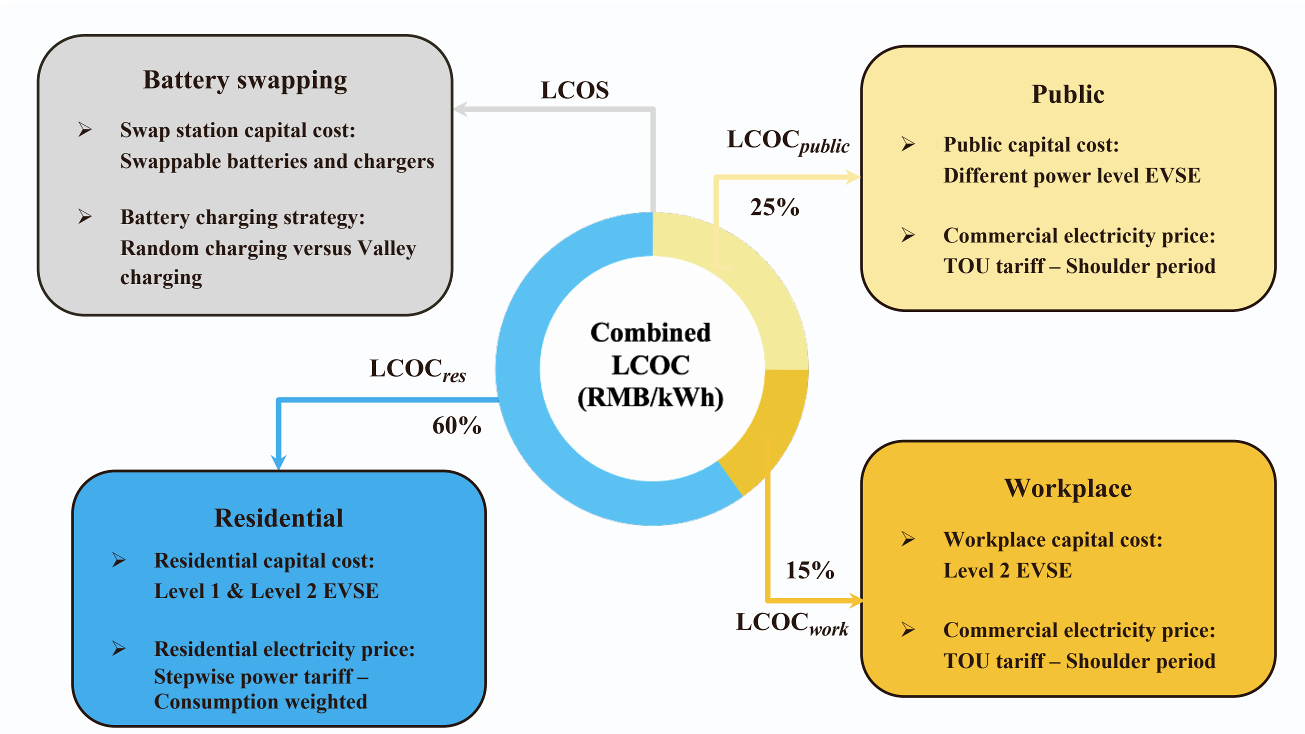

Figure S1. Approach for estimating the combined levelized cost of charging ($LCOC_{comb}$) in China. The baseline with home chargers assumes a charging mix of 60% residential (80% Level 2, 20% Level 1 chargers), 15% workplace, and 25% public charging. Additionally, this study also compares the Levelized Cost of Battery Swapping (LCOBS) to illustrate a comprehensive overview of electric vehicle recharging costs across different scenarios in China [S1] , related to STAR Methods.

S2. Equipment costs of 7 kW Level 2 charging piles

The equipment cost data for our analysis was collected from several online commerce platforms, including Taobao [S2] and Tmall [S3]. In total, we acquire data from 6,248 transactions. The distribution of the collected capital cost data is displayed in Figure S2, with the median value being RMB 1,470. The 5th percentile value is RMB 810, while the 95th percentile value is RMB 3,349.

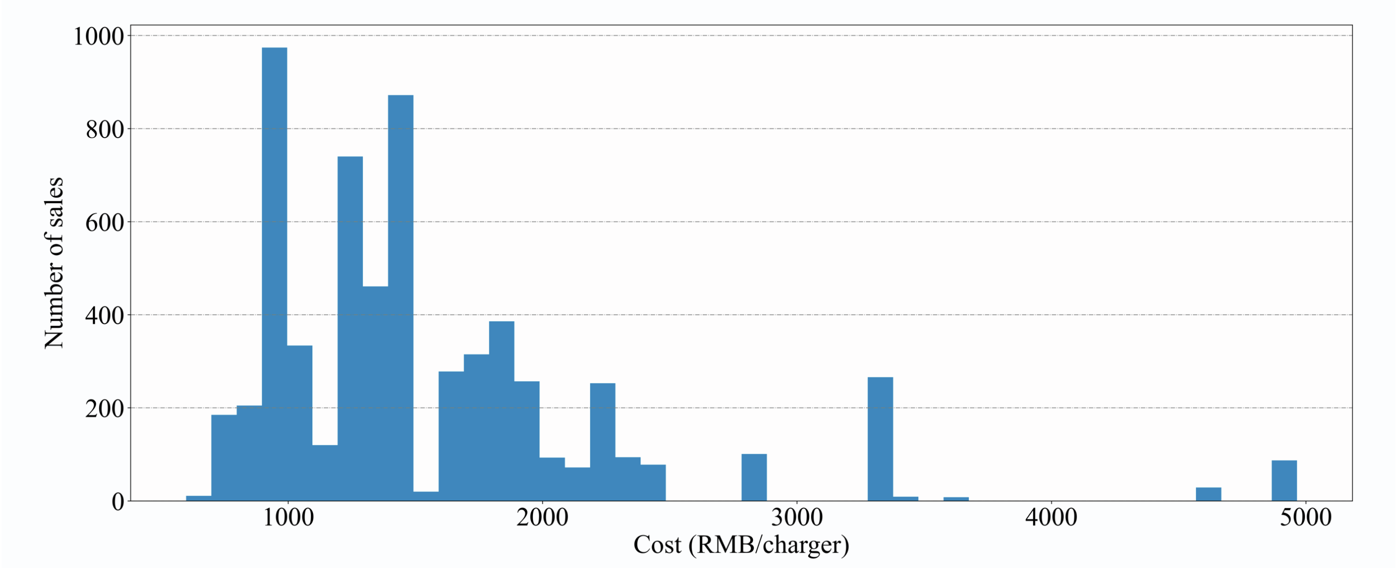

Figure S2. Distribution of the capital costs for 7 kW L2 charging equipment in China, related to STAR Methods.

S3. Consumption-weighted average of residential marginal electricity prices

In China, two types of power tariffs are available for residential customers: Stepwise power tariff (SPT) and Time-of-use (TOU) rates. These tariffs are designed to incentivize residents to adjust their electricity usage in response to varying electricity prices[S4]. Currently, TOU rates are only offered in 20 out of total provinces to encourage customers to shift their electricity consumption to off-peak periods with lower prices. For our study, we use SPT as the baseline case and explore variations in TOU tariffs in the sensitivity analysis.

Under the SPT tariff structure, monthly or annual electricity consumption is divided into different steps, each with a corresponding rate. To calculate the consumption-weighted average residential marginal electricity price for each province ($P_{E,p}$), we utilize Equation S1-S8, taking into account the provincial-level average electricity consumption per resident ($\bar{E}_p$) (Table S1) [S5], the province-specific SPT designs (Table S2), and the number of households of different sizes (*i*) (Table S3) [S5]. The resulting electricity prices for residential charging (E_p) used in our study are presented in Table S4.

$$E_{i,p} = i \times \bar{E}_p \qquad \text{(S1)}$$

where $E_{i,p}$ is the electricity consumption of different sizes of households in each province; i is the size of households in each province. $\bar{E}_p$ is the provincial-level average electricity consumption for a resident (Table S1).

$$N_{l1,p} = \sum N_{i,p} \quad (E_{i,p} \leq S_1) \qquad \text{(S2)}$$

$$N_{l2,p} = \sum N_{i,p} \quad (S_1 \leq E_{i,p} \leq S_2) \qquad \text{(S3)}$$

$$N_{l3,p} = \sum N_{i,p} \quad (E_{i,p} \geq S_2) \qquad \text{(S4)}$$

where $\boldsymbol{N_{l1,p}}$ $\boldsymbol{N_{l2,p}}$ $\boldsymbol{N_{l3,p}}$ are the total number of households in different charging rate level; $\boldsymbol{N_{i,p}}$ is the number of households with different sizes in each province (Table S2); $\boldsymbol{S_1}$ and $\boldsymbol{S_2}$ are the partition step 1 and 2 (Table S3).

$$W_{l1,p} = \frac{N_{l1,p}}{N_{h,p}} \qquad \text{(S5)}$$

$$W_{l2,p} = \frac{N_{l2,p}}{N_{h,p}} \qquad \text{(S6)}$$

$$W_{l3,p} = \frac{N_{l3,p}}{N_{h,p}} \qquad \text{(S7)}$$

where $W_{l1,p}$ $W_{l1,p}$ $W_{l1,p}$ are the provincial-level weights of the number of households in different charging level. $N_{h,p}$ is the total number of households in each province.

$$E_p = W_{l1,p} \times E_{l1,p} + W_{l2,p} \times E_{l2,p} + W_{l3,p} \times E_{l3,p} \qquad \text{(S8)}$$

where $\boldsymbol{E_p}$ is the weighted average marginal electricity price under stepwise power tariff (SPT) structures in each province. $\boldsymbol{E_{l1,p}}$ $\boldsymbol{E_{l2,p}}$ $\boldsymbol{E_{l3,p}}$ are the provincial-level SPT electricity rates (Table S3).

Table S1. Average electricity consumption per person per month [S5], related to STAR Methods.

Province	kWh/month	Province	kWh/month
**Anhui**	51	**Jiangsu**	79
**Beijing**	97	**Jiangxi**	51
**Chongqing**	55	**Jilin**	40
**Fujian**	97	**Liaoning**	54
**Gansu**	34	**Ningxia**	*60
**Guangdong**	78	**Qinghai**	*60
**Guangxi**	62	**Shaanxi**	56
**Guizhou**	*60	**Shandong**	58
**Hainan**	60	**Shanghai**	84
**Hebei**	*60	**Shanxi**	48
**Heilongjiang**	41	**Sichuan**	48
**Henan**	53	**Tianjin**	60
**Hubei**	61	**Xinjiang**	*60
**Hunan**	61	**Yunnan**	42
**Inner Mongolia**	46	**Zhejiang**	91

* Due to the lack of official provincial data, we use the national average instead.

Table S2. The number of households with different sizes at the provincial level in China [S5] , related to STAR Methods.

Province	Total Number of households	Household size							
		1	2	3	4	5	6	7	8
**Anhui**	16243	2,327	4450	3876	2992	1432	826	215	73
**Beijing**	6490	1485	1951	1712	724	444	130	27	11
**Chongqing**	8590	1640	2366	2010	1338	774	359	70	22
**Fujian**	10546	2159	2656	2108	1781	963	615	164	50
**Gansu**	6156	676	1472	1344	1169	720	496	182	55
**Guangdong**	28361	7561	6509	4658	4116	2655	1415	690	326
**Guangxi**	11714	1863	2384	2461	2392	1358	731	281	99
**Guizhou**	8991	1402	2134	1838	1813	967	506	206	66
**Hainan**	2023	266	392	417	481	248	123	53	20
**Hebei**	19580	2431	5924	4506	3644	1683	1006	269	70
**Heilongjiang**	11910	1947	4614	3437	1149	543	174	31	8
**Henan**	22976	2781	5245	4979	4847	2638	1679	547	173
**Hubei**	15269	2511	4280	3794	2419	1322	690	140	56
**Hunan**	16766	2162	4076	4060	3322	1724	938	301	103
**Inner Mongolia**	7852	1109	2743	2562	1030	281	104	17	4
**Jiangsu**	20796	3335	6223	4841	2918	2127	884	271	106
**Jiangxi**	10863	1182	2387	2364	2458	1339	705	273	86
**Jilin**	8305	1313	3034	2370	920	464	161	34	8
**Liaoning**	13791	2663	4949	3943	1353	631	216	30	6
**Ningxia**	1722	204	453	421	355	161	84	29	8
**Qinghai**	1485	267	332	320	269	150	90	35	14
**Shaanxi**	10193	1624	2859	2502	1779	864	410	110	29
**Shandong**	28656	4110	9515	6981	5122	1741	977	168	25
**Shanghai**	7971	1895	2902	1985	697	397	80	12	1
**Shanxi**	10035	1335	2859	2607	2052	761	332	64	20
**Sichuan**	23142	4203	6714	5242	3458	2129	975	300	73
**Tianjin**	4171	614	1349	1361	566	197	66	14	3
**Xinjiang**	6529	890	1579	1668	1380	628	252	85	25
**Yunnan**	11683	1522	2534	2607	2498	1400	788	232	69
**Zhejiang**	17728	4377	6044	3711	1930	1056	471	86	40

Table S3. The existing stepwise power tariff (SPT) systems at the provincial level in China [S4] , related to STAR Methods.

Province	Time period	Partition Step 1 (kWh)	Partition Step 2 (kWh)	Rate below Step 1 (RMB/kWh)	Rate between Step 1 and 2 (RMB/kWh)	Rate above Step 2 (RMB/kWh)
**Anhui**	Monthly	180	350	0.5653	0.6153	0.8653
**Beijing**	Monthly	241	400	0.4883	0.5383	0.7883
**Chongqing**	Annual	2401	4800	0.52	0.57	0.82
**Fujian**	Monthly	161	240	0.4983	0.5483	0.7983
**Gansu**	Monthly	231	421	0.51	0.56	0.81
**Guangdong**	Monthly	261	600	0.68	0.73	0.98
**Guangxi**	Monthly	190	290	0.5283	0.5783	0.8283
**Guizhou**	Annual	2200	4000	0.4556	0.5056	0.7556
**Hainan**	Monthly	220	361	0.6083	0.6583	0.9083
**Hebei**	Monthly	181	280	0.52	0.57	0.82
**Heilongjiang**	Monthly	171	260	0.51	0.56	0.81
**Henan**	Annual	2161	3200	0.56	0.61	0.86
**Hubei**	Annual	2161	4800	0.558	0.608	0.858
**Hunan**	Monthly	201	350	0.588	0.638	0.888
**Inner Mongolia**	Monthly	171	260	0.415	0.465	0.715
**Jiangsu**	Annual	2761	4800	0.5283	0.5783	0.8283
**Jiangxi**	Annual	2161	4800	0.6	0.65	0.9
**Jilin**	Annual	2041	3120	0.525	0.575	0.825
**Liaoning**	Monthly	181	280	0.5	0.55	0.8
**Ningxia**	Annual	2041	3120	0.4486	0.4986	0.7486
**Qinghai**	Monthly	151	230	0.3771	0.4271	0.6771
**Shaanxi**	Annual	2161	4200	0.4983	0.5483	0.7983
**Shandong**	Monthly	211	400	0.5469	0.5969	0.8469
**Shanghai**	Annual	3121	4800	0.617	0.667	0.917
**Shanxi**	Monthly	171	260	0.477	0.527	0.777
**Sichuan**	Monthly	181	280	0.5224	0.6224	0.8224
**Tianjin**	Monthly	221	400	0.49	0.54	0.79
**Xinjiang**	/			0.429		
**Yunnan**	Monthly	171	260	0.45	0.5	0.8
**Zhejiang**	Annual	2761	4800	0.538	0.588	0.838

Note: Xinjiang has no SPT system.

Table S4. The consumption-weighted averages of residential marginal electricity prices under stepwise power tariff (SPT) structures, which are derived from Table S1-3, related to STAR Methods.

Province	Average residential marginal electricity price	Province	Average residential marginal electricity price
**Anhui**	0.588	**Jiangsu**	0.572
**Beijing**	0.536	**Jiangxi**	0.626
**Chongqing**	0.536	**Jilin**	0.530
**Fujian**	0.674	**Liaoning**	0.513
**Gansu**	0.514	**Ningxia**	0.521
**Guangdong**	0.704	**Qinghai**	0.457
**Guangxi**	0.605	**Shaanxi**	0.518
**Guizhou**	0.499	**Shandong**	0.563
**Hainan**	0.644	**Shanghai**	0.640
**Hebei**	0.576	**Shanxi**	0.504
**Heilongjiang**	0.514	**Sichuan**	0.565
**Henan**	0.609	**Tianjin**	0.501
**Hubei**	0.590	**Xinjiang**	0.429
**Hunan**	0.628	**Yunnan**	0.469
**Inner Mongolia**	0.428	**Zhejiang**	0.582

S4. Supplementary figures and tables

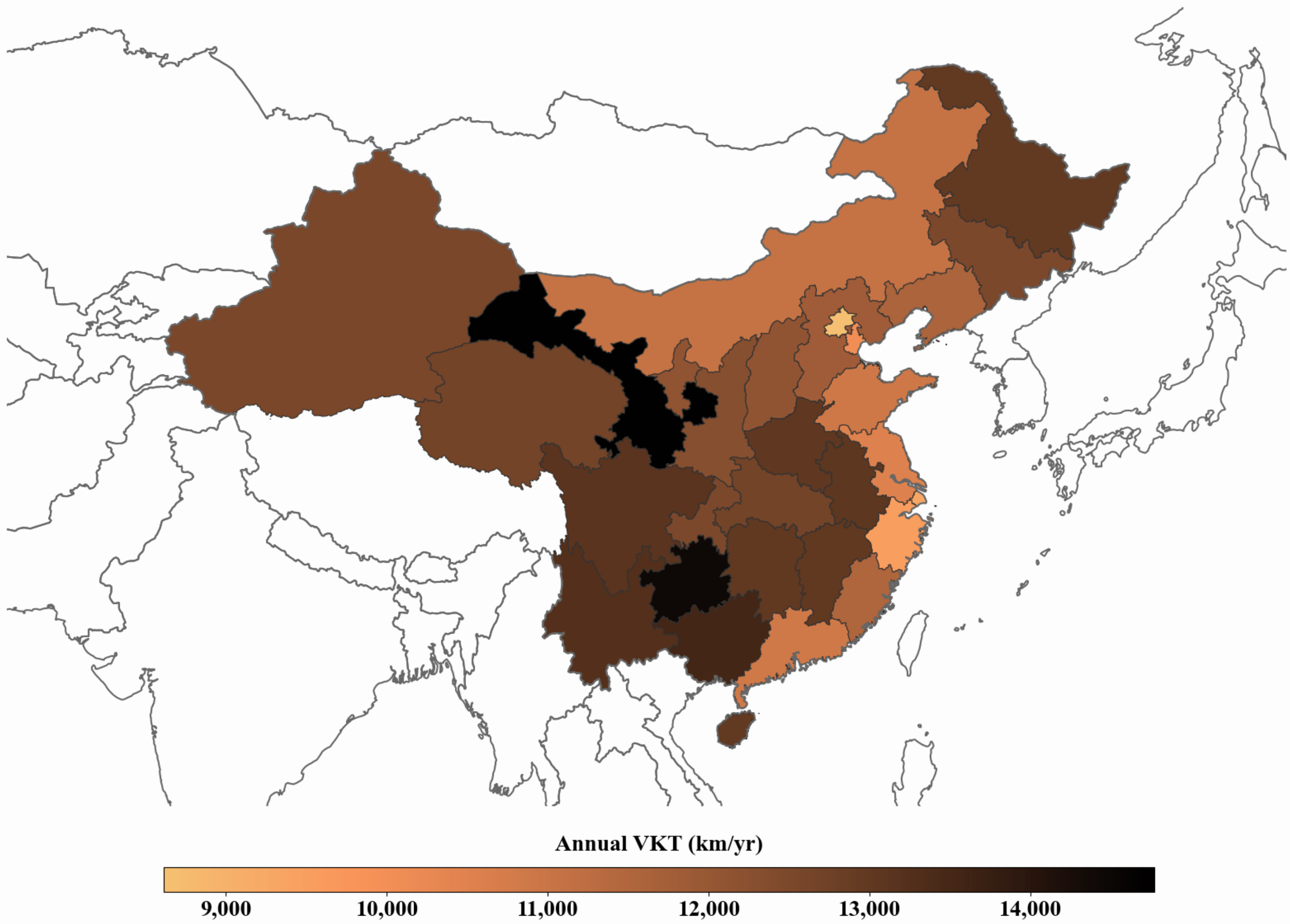

Figure S3. Provincial-level annual vehicle kilometer traveled (VKT) of private passenger cars [S6]. Note that only the provinces of China modeled in the present work are shown using colors, related to STAR Methods.

Table S5. Fuel consumption rate (FCR) of the 2023 top 50 best-selling EV models in China, related to STAR Methods.

EV model	2023 Sales	MIIT FCR (kWh/100km)	EV model	2023 Sales	MIIT FCR (kWh/100km)
**Tesla Model Y**	457617	13.4	**BMW iX3**	39542	15.3
**BYD Seal**	285950	14.7	**VW ID.4 CROZZ**	36693	14.7
**BYD Atto3 plus**	282154	12.3	**BAIC EU**	36198	15.9
**Wuling Mini**	237430	9.2	**Neta U**	34499	13.9
**BYD Seagull**	220638	10.0	**Smart #1**	33149	13.7
**AION.S**	210199	12.9	**LEAPMOTOR T03**	32212	12.5
**AION.Y**	203723	13.3	**Geometry A**	30876	13.2
**Wuling bigove**	163873	10.6	**Geometry E**	30138	11.6
**Tesla Model 3**	147559	12.5	**DFPV E70**	27593	13.3
**Changan Lumin**	142654	10.1	**FAW Toyota BZ3**	23958	11.7
**BYD Qin Plus**	122426	13.0	**CLEVER**	23857	9.9
**Panda Mini**	99133	9.5	**Chery EQ1**	23485	11.6
**BYD Song plus**	88592	15.7	**Xpeng G9**	23290	15.2
**BYD Qin**	76363	13.2	**LEAPMOTOR C01**	23001	13.8
**VW ID.3**	75437	13.1	**VW ID.4 X**	22703	15.8
**Hong Qi E-QM5**	72896	13.1	**Audi Q4 e-tron**	22306	17.5
**BYD Sea I06**	71437	12.7	**Neta V**	22183	10.8
**ORA Cat**	71322	13.3	**AION V**	21212	14.9
**ZEEKR 001**	69180	17.5	**Xpeng P5**	18828	13.3
**Chery QQ**	64695	10.7	**BYD E2**	18565	10.5
**NIO ET5**	62664	16.2	**ZEEKR 009**	18510	18.6
**NIO ES6**	55613	19.1	**Venucir D60**	17833	12.9
**BMW i3**	53745	14.2	**AVATR 11**	17590	16.6
**Xpeng P7**	43355	13.8	**Hycan Z03**	17353	13.5
**Xpeng G6**	42696	13.7	**ZEEKR X**	17093	17.3

Table S6. 2023 average commercial electricity prices under the Time-of-Use rate structure (RMB/kWh) [S4], related to STAR Methods.

Province	Abbreviation	Off-peak	Shoulder	Peak
**Anhui**	AH	0.3274	0.7114	1.2414
**Beijing**	BJ	0.6044	0.8656	1.0722
**Chongqing**	CQ	0.3679	0.7687	1.1900
**Fujian**	FJ	0.4109	0.6815	1.0413
**Gansu**	GS	0.5823	0.6545	0.7294
**Guangdong**	GD	0.3082	0.7758	1.6011
**Guangxi**	GX	0.5060	0.7639	1.1766
**Guizhou**	GZ	0.3505	0.6195	0.8885
**Hainan**	HI	0.3830	0.8609	1.4626
**Hebei**	HE	0.3575	0.6125	0.9916
**Heilongjiang**	HL	0.4029	0.7711	1.2492
**Henan**	HA	0.3391	0.7235	1.2275
**Hubei**	HB	0.3796	0.7883	1.4069
**Hunan**	HN	0.3441	0.7825	1.3232
**Inner Mongolia**	IM	0.3944	0.6540	1.0466
**Jiangsu**	JS	0.3384	0.7489	1.2945
**Jiangxi**	JX	0.3698	0.7100	1.1519
**Jilin**	JL	0.4340	0.8141	1.4222
**Liaoning**	LN	0.3776	0.7109	1.2943
**Ningxia**	NX	0.3709	0.4924	0.6627
**Qinghai**	QH	0.3050	0.4830	0.7859
**Shaanxi**	SN	0.3487	0.6581	1.0212
**Shandong**	SD	0.3016	0.7397	1.2144
**Shanghai**	SH	0.4692	0.8355	0.9894
**Shanxi**	SX	0.3702	0.6239	0.9100
**Sichuan**	SC	0.3176	0.7342	1.1508
**Tianjin**	TJ	0.4238	0.8066	1.1609
**Xinjiang**	XJ	0.2326	0.4392	0.6927
**Yunnan**	YN	0.2720	0.4747	0.6802
**Zhejiang**	ZJ	0.4072	0.8044	1.3592

Table S7. Equipment costs of public EVSE (Unit: RMB)[S7], related to STAR Methods.

Type of equipment	Lower case	Median	Upper case
**50 kW**	15,600	22,690	29,250
**100~120 kW**	22,930	31,136	45,000
**150~175 kW**	33,785	60,809	80,000
**350 kW**	79,920	107,123	125,000

Table S8. Population of provinces in China [S5], related to STAR Methods.

Province	Population	Province	Population
**Anhui**	61,027,171	**Jiangsu**	84,748,016
**Beijing**	21,893,095	**Jiangxi**	45,188,635
**Chongqing**	32,054,159	**Jilin**	24,073,453
**Fujian**	41,540,086	**Liaoning**	42,591,407
**Gansu**	25,019,831	**Ningxia**	7,202,654
**Guangdong**	126,012,510	**Qinghai**	5,923,957
**Guangxi**	50,126,804	**Shaanxi**	39,528,999
**Guizhou**	38,562,148	**Shandong**	101,527,453
**Hainan**	10,081,232	**Shanghai**	24,870,895
**Hebei**	74,610,235	**Shanxi**	34,915,616
**Heilongjiang**	31,850,088	**Sichuan**	83,674,866
**Henan**	99,365,519	**Tianjin**	13,866,009
**Hubei**	57,752,557	**Xinjiang**	25,852,345
**Hunan**	66,444,864	**Yunnan**	47,209,277
**Inner Mongolia**	24,049,155	**Zhejiang**	64,567,588

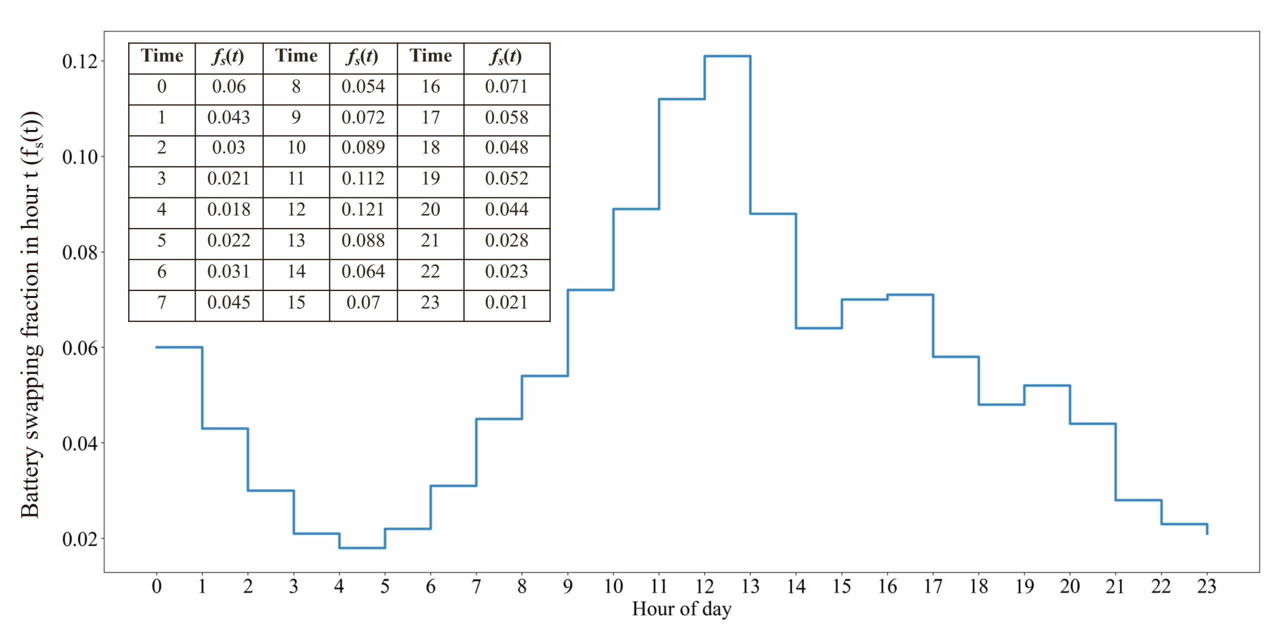

Time	$f_s(t)$	Time	$f_s(t)$	Time	$f_s(t)$
0	0.06	8	0.054	16	0.071
1	0.043	9	0.072	17	0.058
2	0.03	10	0.089	18	0.048
3	0.021	11	0.112	19	0.052
4	0.018	12	0.121	20	0.044
5	0.022	13	0.088	21	0.028
6	0.031	14	0.064	22	0.023
7	0.045	15	0.07	23	0.021

Figure S4. Time distribution of battery swapping demand per day in Beijing [S8], related to STAR Methods.

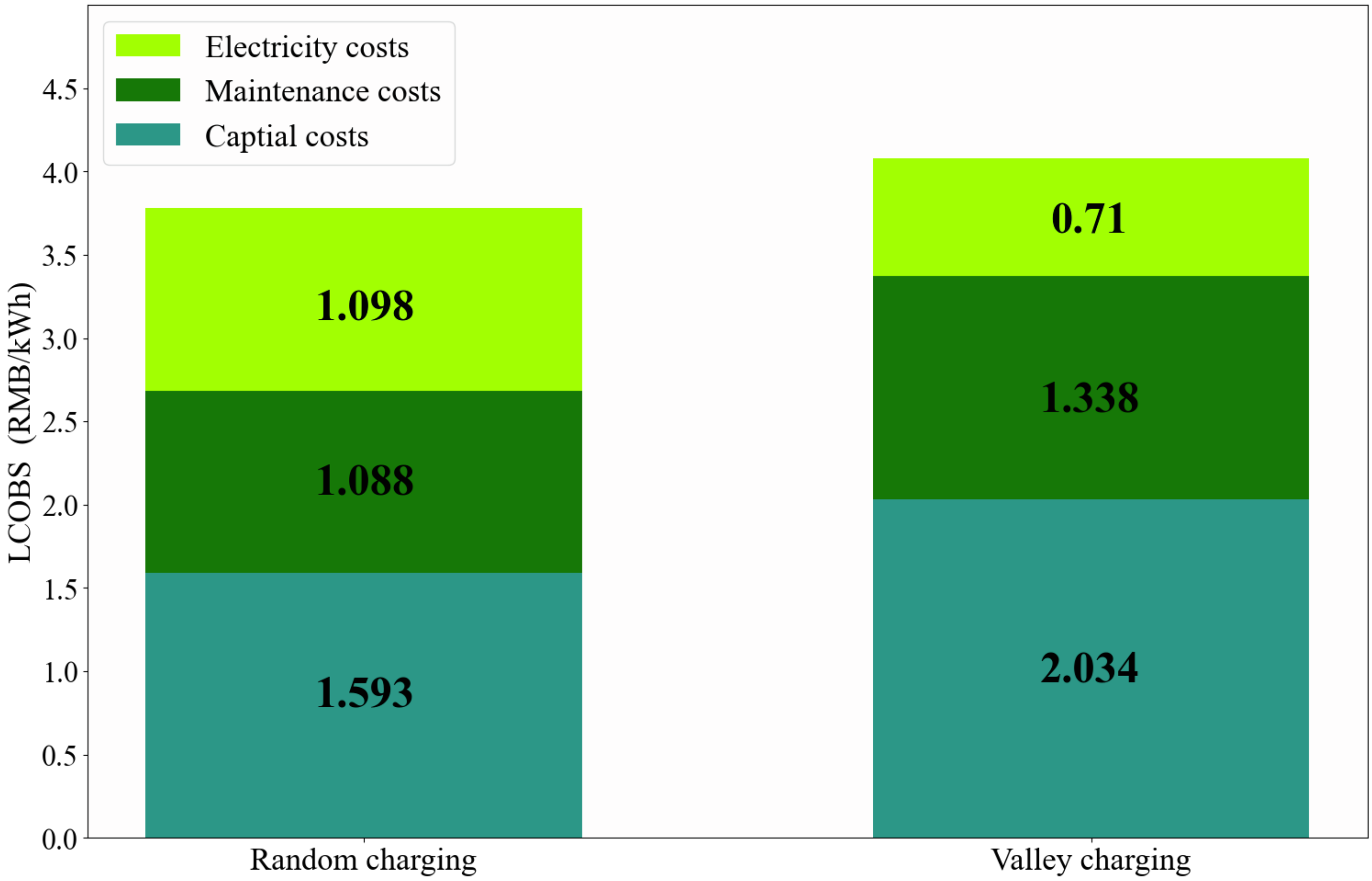

Figure S5. Breakdown of the levelized cost of battery swapping under two different strategies, related to Figure 2.

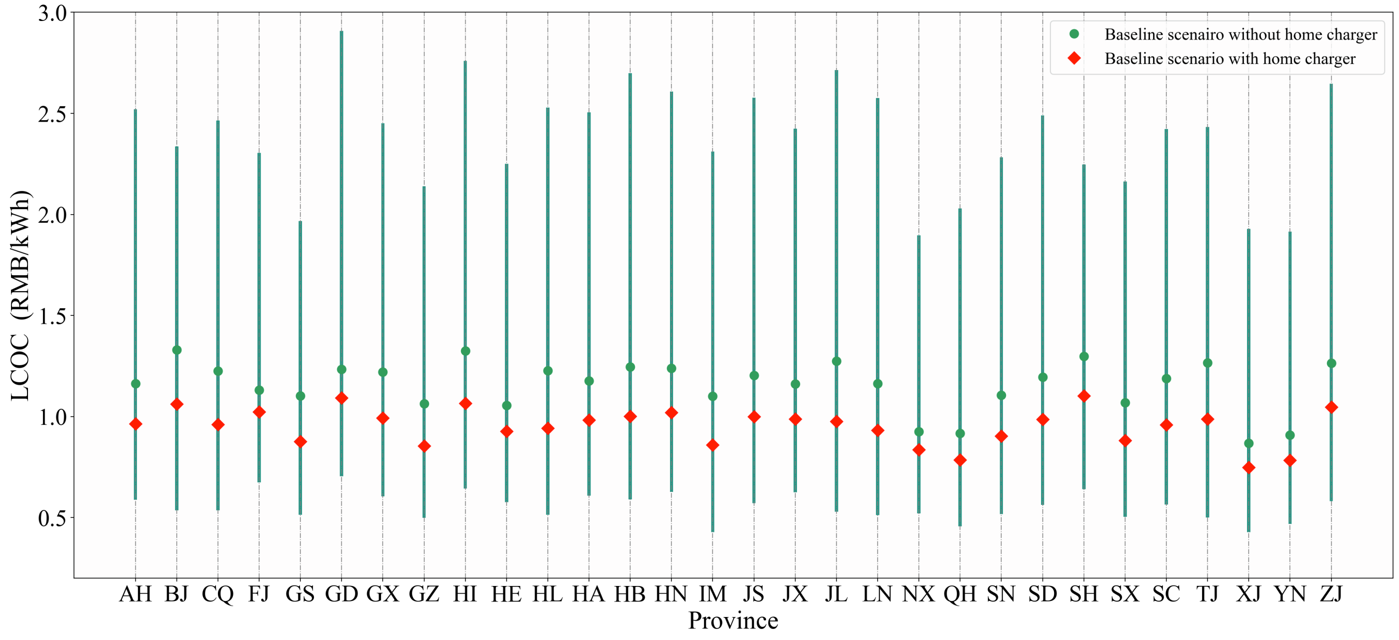

Figure S6. Provincial-level baseline LCOC for EV users with and without home chargers (points), with the compounded sensitivity ranges (bars), related to Figure 2.

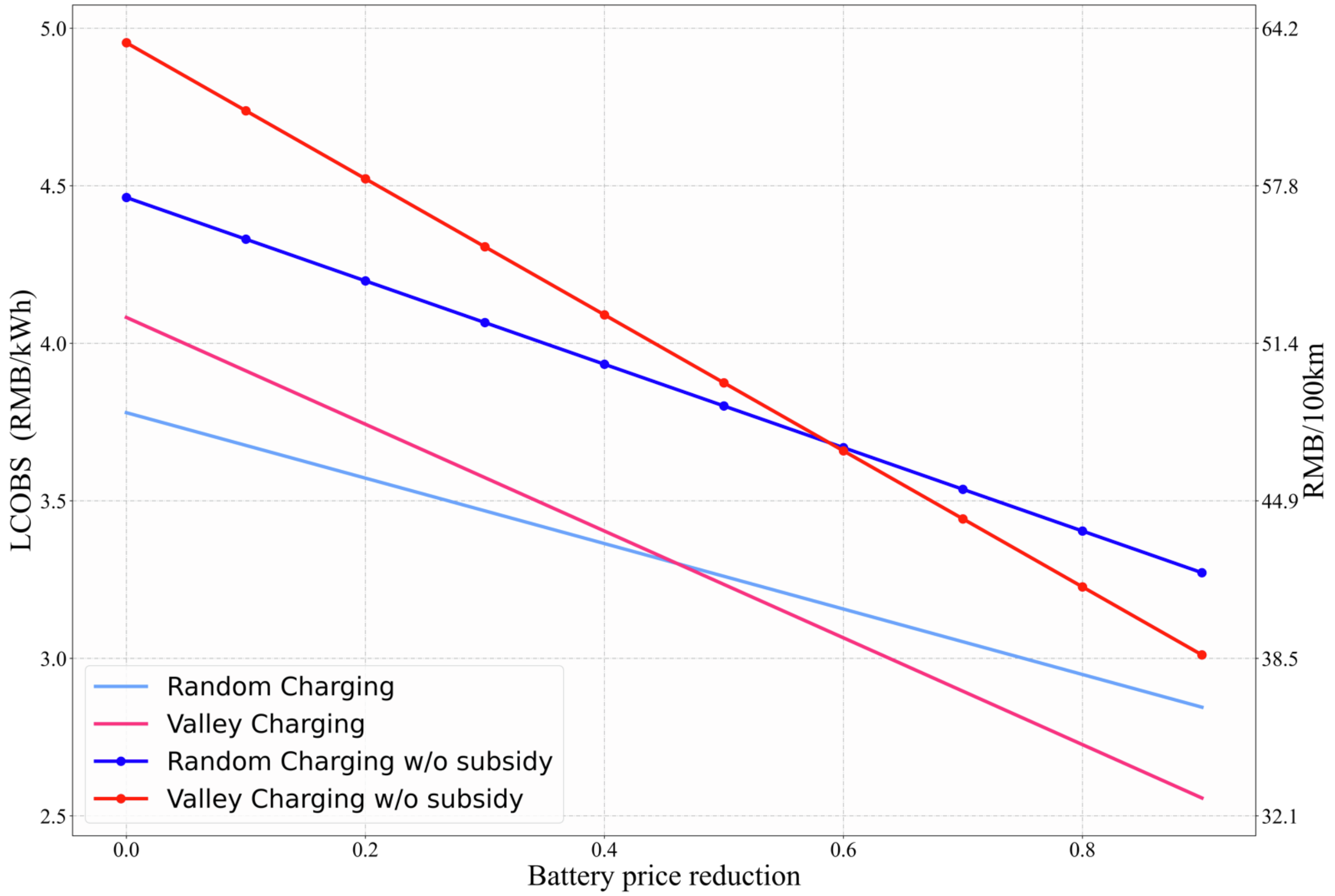

Figure S7. Impacts of battery price reduction with subsidy and without subsidy on LCOBS, related to Figure 3.

Supplemental references

[S1] Ou, S., Lin, Z., He, X., Przesmitzki, S., and Bouchard, J. (2020). Modeling charging infrastructure impact on the electric vehicle market in China. Transp. Res. Part Transp. Environ. 81, 102248. https://doi.org/10.1016/j.trd.2020.102248.

[S2] Taobao (2022). World of Taobao. https://world.taobao.com/.

[S3] Tmall (2022). Tmall. https://www.tmall.com/.

[S4] State Grid Corporation of China (2023). Welcome to State Grid Corporation of China. http://www.sgcc.com.cn/ywlm/index.shtml.

[S5] National Bureau of Statistic (2022). National Bureau of Statistic(国家统计局>>统计数据). http://www.stats.gov.cn/tjsj/.

[S6] Hsieh, I.-Y.L., Chossière, G.P., Gençer, E., Chen, H., Barrett, S., and Green, W.H. (2022). An Integrated Assessment of Emissions, Air Quality, and Public Health Impacts of China's Transition to Electric Vehicles. Environ. Sci. Technol. 56, 6836–6846. https://doi.org/10.1021/acs.est.1c06148.

[S7] Bjx (2020). 2020 Survey of Commercial EV Charger Price (2020 年新能源商用充电桩价格调研). https://news.bjx.com.cn/html/20200303/1049711.shtml.

[S8] Liang, Y., Cai, H., and Zou, G. (2021). Configuration and system operation for battery swapping stations in Beijing. Energy 214, 118883. https://doi.org/10.1016/j.energy.2020.118883.
